# Deciphering the Structural Diversity and Classification of the Mobile Tigecycline Resistance Gene *tet*(X)-Bearing Plasmidome among Bacteria

**DOI:** 10.1128/mSystems.00134-20

**Published:** 2020-04-28

**Authors:** Ruichao Li, Xiaoyu Lu, Kai Peng, Ziyi Liu, Yan Li, Yuan Liu, Xia Xiao, Zhiqiang Wang

**Affiliations:** a College of Veterinary Medicine, Yangzhou University, Yangzhou, Jiangsu, People’s Republic of China; b Jiangsu Co-Innovation Center for Prevention and Control of Important Animal Infectious Diseases and Zoonoses, Yangzhou, Jiangsu, People’s Republic of China; c Institute of Comparative Medicine, Yangzhou University, Yangzhou, Jiangsu, People’s Republic of China; California State University, Fresno

**Keywords:** tigecycline resistance, *tet*(X) variants, tandem repeats, resistance plasmidome, Nanopore sequencing

## Abstract

The emergence of novel plasmid-mediated resistance genes constitutes a great public concern. Recently, mobile *tet*(X) variants were reported in diverse pathogens from different sources. However, the diversity of *tet*(X)-bearing plasmids remains largely unknown. In this study, the phenotypes and genotypes of all the *tet*(X)-positive tigecycline-resistant strains isolated from a slaughterhouse in China were characterized by antimicrobial susceptibility testing, conjugation, pulsed-field gel electrophoresis with S1 nuclease (S1-PFGE), and PCR. The diversity and polymorphism of *tet*(X)-harboring strains and plasmidomes were investigated by whole-genome sequencing (WGS) and single-plasmid-molecule analysis. Seventy-four *tet*(X4)-harboring Escherichia coli strains and one *tet*(X6)-bearing Providencia rettgeri strain were identified. The *tet*(X4)-bearing elements in 27 strains could be transferred to the recipient strain via plasmids. All *tet*(X4)-bearing plasmids isolated in this study and 15 *tet*(X4)-bearing plasmids reported online were analyzed. *tet*(X4)-bearing plasmids ranged from 9 to 294 kb and were categorized as ColE2-like, IncQ, IncX1, IncA/C2, IncFII, IncFIB, and hybrid plasmids with different replicons. The core *tet*(X4)-bearing genetic contexts were divided into four major groups: IS*CR2*-*tet*(X4)-*abh*, △IS*CR2*-*abh*-*tet*(X4)-IS*CR2*, IS*CR2-abh*-*tet*(X4)-IS*CR2*-*virD2*-*floR*, and *abh*-*tet*(X4)-IS*CR2*-*yheS*-*cat*-*zitR*-IS*CR2*-*virD2*-*floR*. Tandem repeats of *tet*(X4) were universally mediated by IS*CR2*. Different *tet*(X)-bearing strains existed in the same microbiota. Reorganization of *tet*(X4)-bearing multidrug resistance plasmids was found to be mediated by IS*26* and other homologous regions. Finally, single-plasmid-molecule analysis captured the heterogenous state of *tet*(X4)-bearing plasmids. These findings significantly expand our knowledge of the *tet*(X)-bearing plasmidome among microbiotas, which establishes a baseline for investigating the structure and diversity of human, animal, and environmental tigecycline resistomes. Characterization of *tet*(X) genes among different microbiotas should be performed systematically to understand the evolution and ecology.

**IMPORTANCE** Tigecycline is an expanded-spectrum tetracycline used as a last-resort antimicrobial for treating infections caused by superbugs such as carbapenemase-producing or colistin-resistant pathogens. Emergence of the plasmid-mediated mobile tigecycline resistance gene *tet*(X4) created a great public health concern. However, the diversity of *tet*(X4)-bearing plasmids and bacteria remains largely uninvestigated. To cover this knowledge gap, we comprehensively identified and characterized the *tet*(X)-bearing plasmidome in different sources using advanced sequencing technologies for the first time. The huge diversity of *tet*(X4)-bearing mobile elements demonstrates the high level of transmissibility of the *tet*(X4) gene among bacteria. It is crucial to enhance stringent surveillance of *tet*(X) genes in animal and human pathogens globally.

## Abstract (precis)

Tigecycline is an expanded-spectrum tetracycline used as a last-resort antimicrobial for treating infections caused by superbugs such as carbapenemase-producing or colistin-resistant pathogens. Emergence of the plasmid-mediated mobile tigecycline resistance gene *tet*(X4) created a great public health concern. However, the diversity of *tet*(X4)-bearing plasmids and bacteria remains largely uninvestigated. To cover this knowledge gap, we comprehensively identified and characterized the *tet*(X)-bearing plasmidome in different sources using advanced sequencing technologies for the first time. The huge diversity of *tet*(X4)-bearing mobile elements demonstrates the high level of transmissibility of the *tet*(X4) gene among bacteria. It is crucial to enhance stringent surveillance of *tet*(X) genes in animal and human pathogens globally.

## INTRODUCTION

Antibiotics, considered a major breakthrough of modern medicine, are drugs utilized to treat bacterial infections in humans and animals (1, 2). However, the emergence of antibiotic resistance (AR) is becoming a great threat to human and animal health worldwide, to a great extent resulting from misuse, abuse, and overuse of antibiotics (2). Furthermore, environmental contamination with antibiotic resistance genes (ARG) is a contributor to AR among pathogens (3, 4). Owing to the complex facilitators of AR transmission in humans, animals, and environments, the One Health approach was proposed to tackle the expanding AR problem globally (5). The presence of multidrug-resistant (MDR) bacteria in animal fecal microbiotas as a factor in accelerating their transmission has been recognized, with a focus on the resistome derived from animal and environmental microbiomes (6). The structural diversity of resistance plasmids derived from fecal microbiotas has scarcely been investigated.

Tetracycline antibiotics have been a fundamental antibacterial agent for more than 6 decades and are widely used in clinical settings and animal sectors because of (i) their broad-spectrum activity against Gram-positive, Gram-negative, and atypical bacteria, (ii) their low cost, (iii) and their ability to be administered orally and intravenously (7–10). Tetracyclines exert antimicrobial activity by inhibiting bacterial protein synthesis through binding to the 30S bacterial ribosome subunit (11). Due to the extensive usage of tetracyclines, resistance has emerged among commensal bacteria and pathogens via two major mechanisms, including efflux pumps and ribosome protection, and is now widespread (9, 12). To counter tetracycline resistance, tigecycline, a semisynthetic glycylcycline derivative of tetracycline, was approved for clinical use in 2005 (9). With the emergence and spread of carbapenemase-producing *Enterobacteriaceae* (CRE) and colistin-resistant *Enterobacteriaceae* (13, 14), tigecycline is regarded as the last-resort antibiotic to treat severe infections caused by MDR pathogens. Although tigecycline can evade bacterial tetracycline resistance posed by drug efflux pumps and ribosomal protection (15), resistance to tigecycline has been reported and is caused by upregulation of efflux pumps or mutations (16–20). Another tigecycline resistance mechanism can be conferred by a flavin-dependent monooxygenase, Tet(X), which can inactivate tigecycline enzymatically (21, 22). Although *tet*(X) was first discovered in the obligate anaerobe Bacteroides fragilis (23), the emergence of *tet*(X) and its variants in clinical pathogenic microbiotas constitutes a potential public health risk (24–26). Environmental microbiota analyses indicated that *tet*(X) existed in environmental bacteria such as *Flavobacterium* and *Bacteroides* (27–29), which implied that environmental microbiotas may be one source of *tet*(X). However, the horizontal gene transfer (HGT) of *tet*(X) among microbiotas, especially those most closely related to human health, was not investigated in detail.

Recently, two reports highlighted the emergence of plasmid-mediated *tet*(X3) and *tet*(X4) conferring high-level tigecycline resistance in bacteria of animal, food, and human origins (30, 31). This indicates that HGT of mobile tigecycline resistance *tet*(X) genes among pathogens is becoming a real threat, which is further demonstrated by the emergence of *tet*(X) genes from different sources, novel *tet*(X5) in Acinetobacter baumannii and *tet*(X6) in *Proteus* spp. (32–38). Various *tet*(X4)-bearing plasmids have been characterized (30, 31, 35, 39, 40), but the diversity and polymorphism of *tet*(X4)-bearing genetic structures, especially in the tigecycline resistome of animal fecal microbiotas and surroundings, was not investigated systematically. In this study, we probed the diversity and polymorphism of *tet*(X4)-bearing plasmids genomically and phenotypically, with the perspective of the resistance plasmidome. The findings imply that the mobile tigecycline resistance gene *tet*(X4) is present extensively in fecal commensals and environments.

## RESULTS AND DISCUSSION

### Identification of *tet*(X)-positive strains and resistance phenotypes.

Among 240 samples, 68 (28.33%) yielded 75 *tet*(X)-positive strains, consisting of 74 Escherichia coli strains harboring *tet*(X4) and 1 Providencia rettgeri strain harboring *tet*(X6) (RF14-2), confirmed by 16S rRNA gene sequencing and Sanger sequencing of PCR products. The rates of isolation from wastewater and soil samples were the highest (40%), followed by swine feces and ground blood samples (see Table S1 in the supplemental material). The average prevalence of *tet*(X)-bearing samples was 28.33%, higher than that in two previous reports (30, 31). This indicated that *tet*(X)-positive strains, especially *tet*(X4)-bearing E. coli strains, existed in slaughterhouse environments at high prevalence and probably resulted from contamination from imported *tet*(X4)-bearing fecal microbiotas. Previously, E. coli strains from farm pigs and pork samples in markets were found positive for *tet*(X4), implying that slaughterhouse processing of pigs was a vital procedure for controlling *tet*(X4) contamination (35, 41). Notably, two *tet*(X)-positive strains were recovered from the sample among seven samples (see Table S2), demonstrating that *tet*(X) genes could be transmitted within the same microbiota. All the *tet*(X4)-positive strains and one *tet*(X6)-positive strain exhibited resistance to tigecycline, with MICs from 8 to 32 mg/liter. In addition, all the strains were resistant to tetracycline and florfenicol, with a high rate of resistance to amoxicillin, doxycycline, and streptomycin, and most of them were multidrug-resistant strains. However, all strains were still susceptible to colistin and meropenem, except Providencia rettgeri RF14-2, which had intrinsic resistance to colistin (see Table S2).

10.1128/mSystems.00134-20.4 TABLE S1 Numbers of *tet*(X4)-positive strains and their prevalence among different sources in the slaughterhouse. Download Table S1, DOCX file, 0.01 MB. Copyright © 2020 Li et al. 2020 Li et al. This content is distributed under the terms of the Creative Commons Attribution 4.0 International license.

10.1128/mSystems.00134-20.5 TABLE S2 Antibiotic susceptibility testing (in milligrams per liter) of 74 E. coli strains harboring *tet*(X4) and 1 Providencia rettgeri strain harboring *tet*(X6). Download Table S2, DOCX file, 0.03 MB. Copyright © 2020 Li et al. 2020 Li et al. This content is distributed under the terms of the Creative Commons Attribution 4.0 International license.

### Genomic epidemiology of *tet*(X4)-carrying E. coli isolates.

**FIG 1:**
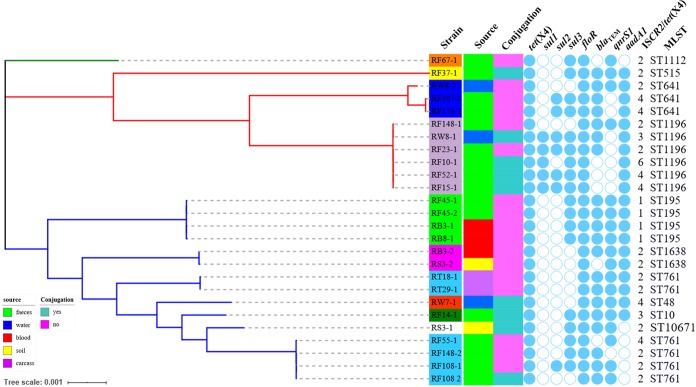
Phylogenetic tree of 26 *tet*(X4)-positive E. coli isolates from different sources and their basic characterization. Three clusters (branches are in green, red, and blue) were identified. Strain IDs with different-colored backgrounds correspond to STs. The genes *floR* and IS*CR2* were found in all 26 *tet*(X4)-positive strains.

Twenty-seven strains, including twenty-six E. coli isolates and one Providencia rettgeri isolate from different sources, were randomly selected for whole-genome sequencing (WGS) with the Illumina HiSeq 2500 platform. These strains were isolated from different sources, including feces, wastewater, blood, soil, and carcasses. Draft genomes of these isolates were generated via *de novo* assembly for subsequent analysis. To investigate the evolutionary relatedness of these 26 E. coli strains, a phylogenetic tree based on single nucleotide polymorphisms (SNPs) of core genomes was constructed (Fig. 1). The phylogenetic tree showed that the 26 E. coli strains were grouped into three clusters. Multilocus sequence typing (MLST) analysis revealed that the 26 E. coli strains were assigned to nine known MLST types, with ST1196 being the most prevalent type, and strain RS3-1 belonged to a novel ST, designated ST10671 by Enterobase (42). The phylogenetic tree and diversity of MLST types showed that the *tet*(X4)-carrying E. coli strains isolated from swine feces and environmental samples in this slaughterhouse were diverse and had no obvious clonal spread. Other STs of E. coli, such as ST8302, ST101, and ST542, were positive for *tet*(X4) (31, 35). The wide distribution of ST numbers of *tet*(X4)-bearing E. coli strains indicated that horizontal gene transfer was the major transmission route for *tet*(X4) among E. coli strains, although *tet*(X4) was occasionally found on the chromosome (41, 43). The 26 *tet*(X4)-bearing strains were positive for at least three categories of resistance genes, with the most prevalent being *floR*, *aadA1*, *bla*_TEM_, and *qnrS1* (Fig. 1), which indicates that *tet*(X4) has a risk of cotransmission with other resistance genes.

### Transmissibility of *tet*(X4)-bearing genetic structures.

To investigate the transmissibility of the *tet*(X4) and *tet*(X6) genes and their genetic contexts, all 75 strains were subjected to conjugation assay. The *tet*(X4) gene from 27 isolates and the corresponding resistance phenotype were successfully transferred to E. coli C600, suggesting that the 27 isolates harbored the *tet*(X4)-positive genetic structures in conjugative plasmids or other mobilizable genetic elements. Plasmid fingerprints of *tet*(X4)-positive strains and corresponding transconjugants resolved by pulsed-field gel electrophoresis with S1 nuclease (S1-PFGE) were utilized to probe the plasmid profiles. All the donor strains harbored one to three plasmids, and at least one plasmid existed in transconjugants, which indicated that the *tet*(X4) gene was in self-conjugative or mobilizable plasmids, and other plasmids could be cotransferred to the recipient strain. Notably, the plasmids of six transconjugants were larger than the plasmids of their donor strains, implying that plasmid reorganizations may occur. The genetic basis of the *tet*(X4)-bearing plasmids and plasmid reorganizations was investigated further.

### A wide variety of *tet*(X4)-harboring plasmids.

According to S1-PFGE, a total of 27 *tet*(X4)-harboring plasmids with different sizes were selected and sequenced with the long-read Nanopore MinION platform, and these plasmids ranged from 12 to 294 kb in length (see Table S3). The majority of *tet*(X4)-harboring plasmids were *de novo* assembled completely by combining short-read and long-read data, and a few of them were *de novo* assembled based on available long-read data. Sixteen plasmids could be successfully transferred into the recipient E. coli C600. These 27 *tet*(X4)-harboring plasmids were categorized into nine different replicon types. Eight plasmids, ranging from 97 to 129 kb, belonged to IncFIB(K)/IncFIA(HI1)/IncX1 group of hybrid plasmids. Four plasmids, ranging from 112 to 136 kb, were classified as IncFII-type plasmids. Three plasmids, ranging from 31 to 50 kb, were classified as IncX1-type plasmids. The rest of the plasmids ranged from 12 to 294 kb and were classified as IncQ1 type, IncA/C2 type, IncFIB type, IncFIA(HI1)/IncHI1A/IncHI1B(R27) hybrid type, IncHI1B(R27)/IncFIA(HI1)/IncHI1A/IncX1 hybrid type, and IncFIB(K)/IncFIA(HI1)/IncHI1A/IncHI1B(R27) hybrid type (from small to large) (see Table S3). In total, nine types of *tet*(X4)-harboring plasmids were detected in the same slaughterhouse. These types of plasmids were more abundant than the existing 15 *tet*(X4)-bearing plasmids of different replicons in the NCBI database (see Table S4), which indicated that the microbiome in slaughterhouse had become a reservoir of *tet*(X4). In addition, most of these *tet*(X4)-harboring plasmids were conjugative, showing that the plasmid-mediated trait greatly enhanced transmissibility of the tigecycline resistance gene *tet*(X4).

10.1128/mSystems.00134-20.6 TABLE S3 Basic information on 27 *tet*(X4)-bearing plasmids in this study. Download Table S3, DOCX file, 0.02 MB. Copyright © 2020 Li et al. 2020 Li et al. This content is distributed under the terms of the Creative Commons Attribution 4.0 International license.

10.1128/mSystems.00134-20.7 TABLE S4 Basic information on 42 *tet*(X4)-bearing plasmids in the NCBI nr database and this study. Download Table S4, DOCX file, 0.03 MB. Copyright © 2020 Li et al. 2020 Li et al. This content is distributed under the terms of the Creative Commons Attribution 4.0 International license.

**FIG 2:**
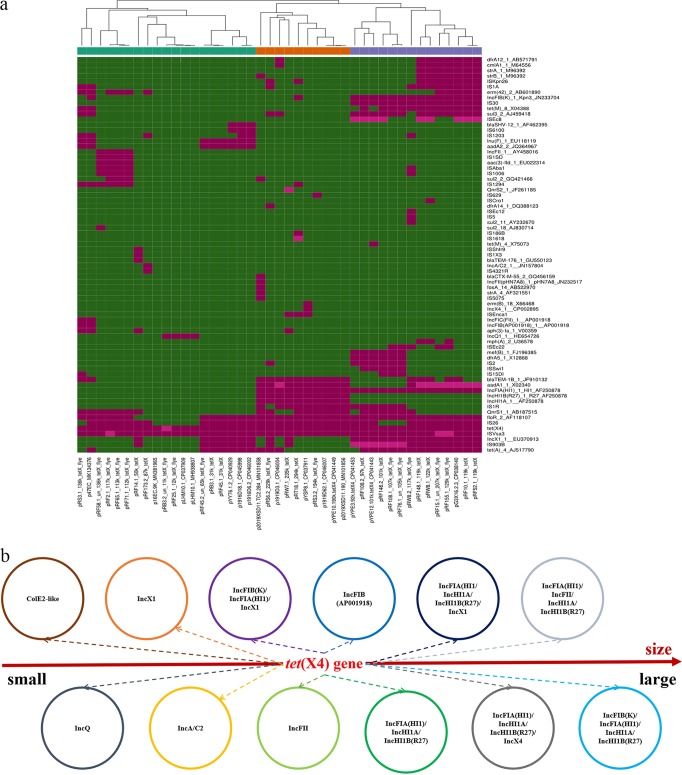
Characterization of 42 *tet*(X4)-bearing plasmids comprising plasmids in the publicly accessible database and in this study. (a) Phylogenetic tree showing distribution of resistance genes and other elements of the *tet*(X4)-bearing plasmids. Red denotes the presence of corresponding genes, and green represents negative results. (b) Diversity of *tet*(X4)-bearing plasmids in terms of replicon types and sizes. For detailed information on the plasmids, refer to Table S4.

**FIG 3:**
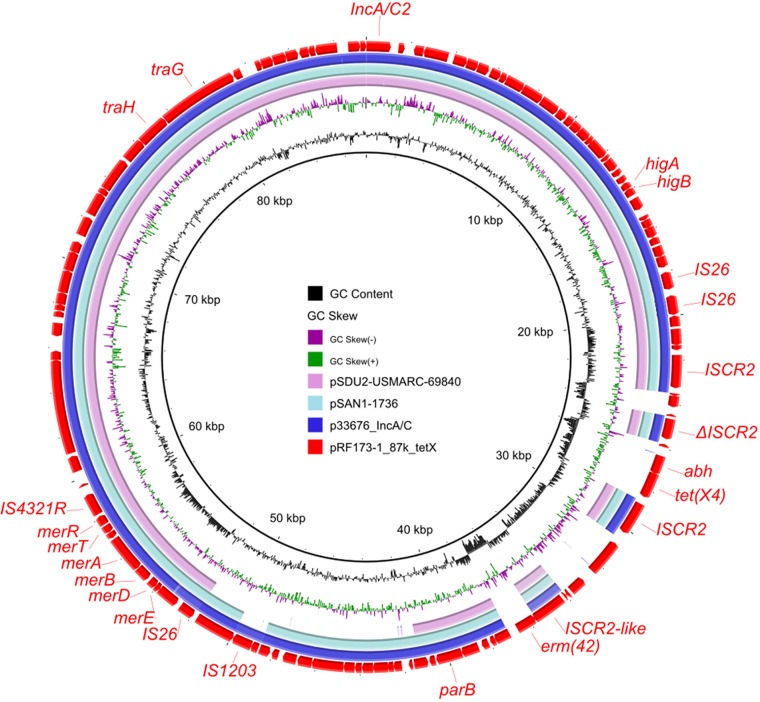
Circular comparison between the *tet*(X4)-bearing IncA/C2 plasmid pRF173-1_87k_tetX and other IncA/C plasmids in the NCBI nr database. The novel *tet*(X4)-bearing IncA/C2 plasmid pRF173-1_87k_tetX was used as the reference in the outermost ring.

To investigate the characterization of all available *tet*(X4)-bearing plasmids, 15 *tet*(X4)-bearing plasmids from the NCBI nonredundant (nr) database were retrieved as of 1 December 2019 (see Table S4). These were analyzed with the 27 *tet*(X4)-bearing plasmids sequenced in this study to determine the distribution of resistance genes, replicons, and insertion sequences among the 42 plasmids (Fig. 2). It was found that IS*CR2* was associated with *tet*(X4) in all plasmids, no matter what the plasmid types were. This observation was consistent with the idea that IS*CR2* could mediate the generation of the circular form, facilitating transmission of *tet*(X4) between different genetic contexts (30, 43). IS*CR2* had already been reported to be associated with various resistance genes, such as *sul2* and *floR* (44). The resistance gene *floR*, conferring resistance to florfenicol, which is often used in animal settings, was the second most closely associated gene with *tet*(X4), being absent from only four plasmids, i.e., three IncQ plasmids and one IncA/C2 plasmid (Fig. 2a). To the best of our knowledge, the IncA/C2 plasmid pRF173-1_87k_tetX characterized in this study is a novel *tet*(X4)-bearing plasmid. It is 87,445 bp in size and carries genes related to plasmid replicon, maintenance, conjugative elements, and resistance, including *erm*(42) and *tet*(X4) flanked by IS*CR2* and IS*26*. Online BLASTn analysis showed that it was most similar to the online IncA/C plasmids found in *Salmonella* spp. and other *Enterobacteriaceae* at 88% coverage with more than 99% identity (Fig. 3). IncA/C plasmids are large, present in low copy numbers, conjugative, and associated with the emergence of multidrug resistance in enteric pathogens of humans and animals (45). IS*CR* elements, including IS*CR2*, are key players in IncA/C plasmid evolution (46), and the emergence of the *tet*(X4)-bearing IncA/C2 plasmid may be mediated by IS*CR2* and the circular intermediate IS*CR2*-*tet*(X4)-*abh* detected previously (30). The occurrence of *tet*(X4) in this classical MDR plasmid would draw wide attention, and surveillance on *tet*(X4)-bearing IncA/C2 plasmids in environmental microbiotas and pathogens should be performed.

The smallest *tet*(X4)-bearing plasmid identified in this study was pRF25-1_12k_tetX_flye, belonging to IncQ1, which was most similar to pLHM10-1-p6, with the genetic structure IS*CR2*-*catD*-*tet*(X4)-△IS*CR2* (see Fig. S1a) (31). IncQ plasmids are a large group of small mobilized plasmids (5.1 to 14.2 kb) with wide host ranges carrying various resistance genes (47, 48). Their mobilization takes place with the help of other conjugative plasmids, which was proved by the cotransmission of pRF25-1_12k_tetX_flye and pRF25-1_147k_flye, an IncFIB/IncX1 hybrid conjugative plasmid harboring *mph*(A), *aac(3)-VIa*, *aadA1*, *tet*(M), *qnrS1*, *bla*_TEM-1A_, and *floR*. IncQ plasmids were found distributed in environmental microbiomes such as wastewater (49), provoking the concern that *tet*(X4)-bearing IncQ plasmids may exist in other environmental microbiomes.

10.1128/mSystems.00134-20.1 FIG S1 Circular comparison of different *tet*(X4)-bearing plasmids with similar online plasmids. a, b, c, d, e, and f represent different *tet*(X4)-bearing plasmids with similar backbones. Download FIG S1, JPG file, 0.8 MB. Copyright © 2020 Li et al. 2020 Li et al. This content is distributed under the terms of the Creative Commons Attribution 4.0 International license.

**FIG 4:**
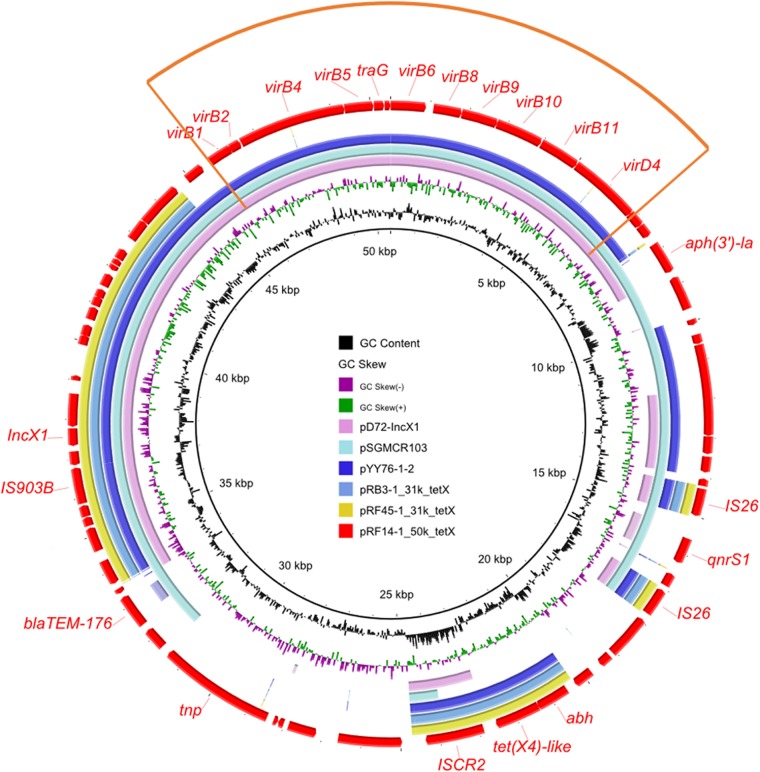
Circular comparison between *tet*(X4)-bearing IncX1 plasmids found in this study and in the online database. The *tet*(X4)-bearing IncX1 plasmid pRF14-1_50k_tetX was used as the reference in the outermost ring, with the orange sector depicting the type IV secretion system (T4SS) region. Lack of T4SS would lead to loss of conjugative ability of IncX1 plasmids.

Three IncX1 plasmids were also found among these 27 plasmids. In the NCBI database, some *mcr*-harboring plasmids and *tet*(X4)-harboring plasmids belonging to the IncX1 type and sharing similar backbones were found (39, 50) (Fig. 4). The *tet*(X4)-harboring IncX1 plasmids ranged from 31 to 59 kb and were characterized by two variable regions, including a multidrug resistance region and a type IV secretion system (T4SS) gene cluster containing different *virB* genes (Fig. 4). Two plasmids, pRB3-1_31k_tetX and pRF45-1_31k_tetX, with deletion of the T4SS gene cluster lost the conjugative ability. Owing to the existence of *tet*(X4)-harboring and *mcr-1*-harboring IncX1 plasmids, the co-occurrence of *tet*(X4) and *mcr-1* in the same conjugative IncX1 plasmid should be put under close surveillance for risk assessment.

According to *tet*(X4)-bearing plasmid type distribution, IncFIB(K)/IncFIA(HI1)/IncX1 hybrid plasmids were the most widespread in the sequenced plasmids. This type of plasmid was also found in plasmids deposited in the online database (see Table S4). They were clustered into two lineages in the phylogenetic tree (Fig. 2a). BLASTn comparison of these IncFIB(K)/IncFIA(HI1)/IncX1 hybrid plasmids with those in the NCBI database showed more than 95% nucleotide identity at 80% coverage to two other *tet*(X4)-bearing plasmids, pYPE12-101k-tetX4 (CP041443.1) and pG3X16-2-3 (CP038140.1). Among those plasmids, pRF10-1_119k_tetX, pRW8-1_122k_tetX, pRW8-2_117k_tetX_flye, and pRF155_129k_tetX_flye showed a backbone similar to that of pG3X16-2-3 (see Fig. S1b), belonging to the first lineage harboring resistance genes, including *qnrS1*, *mef*(B), and *dfrA5* (Fig. 2a); pRF108-1_107k _tetX_flye and pRF148-2_101k_tetX showed a backbone similar to that of pYPE12-101k-tetX4 (see Fig. S1c), belonging to the second lineage harboring resistance genes, including *strAB*, *dfrA12*, *cmlA1*, and *aadA1* (Fig. 2a). Based on the conjugation assay, most of these hybrid plasmids were conjugative, but a few of them could not be transferred successfully. The underlying mechanism warrants further study.

Four IncFII plasmids (pRF58-1_un_136k_tetX_flye, pRF2-1_117k_tetX_flye, pRF65-1_113k_tetX_flye, and pRF71-1_112k_tetX_flye) were identified in these 27 plasmids (see Table S3). BLASTn analysis of the four plasmids in NCBI database showed that they had 95% nucleotide identity at more than 70% coverage to p47EC (MK134376.1), which was the first identified *tet*(X4)-bearing plasmid in E. coli (30) (see Fig. S1d). Meanwhile, another IncFIB type plasmid, pRS3-1_136k_tetX_flye, also shared a similar backbone with p47EC (see Fig. S1d). Even though the backbones of the five plasmids were similar to that of p47EC (all of them belonged to the major IncF incompatibility type), they belonged to different IncF replicons compared with the plasmid p47EC (IncFIB).

The rest of *tet*(X4)-bearing plasmids belonged to different incompatibility types (Fig. 2b; also, see Table S4 and Fig. S1). Apart from the *tet*(X4)-bearing plasmids, a *tet*(X6)-bearing integrative and conjugative element (ICE) was found in RF14-2, which is discussed below.

### Two *tet*(X4)-positive strains were detected in some samples.

Interestingly, two different *tet*(X)-positive strains were detected in the same sample for seven samples (see Table S5). These strains were designated RB3-1, RB3-2, RS3-1, RS3-2, RW8-1, RW8-2, RF148-1, RF148-2, RF14-1, RF14-2, RF108-1, RF108-2, RF45-1, and RF45-2 and were divided into seven groups according to the sources. A single sample containing multiple *tet*(X4)-positive isolates suggested that *tet*(X4) had spread in the same microbiota. We also found that the *tet*(X4)-harboring plasmids carried by these strains were diverse (see Table S5). Meanwhile, WGS analysis showed that strains RF108-1 and RF108-2 are similar phylogenetically to each other and that RF45-1 and RF45-2 have a common ancestor (Fig. 1). Copy number variations of the *tet*(X4) gene were found between *tet*(X4)-harboring plasmids in strains RF45-1 and RF45-2 (see Table S3). A helper plasmid was detected in strain RF108-2 but absent from strain RF108-1, and it played an important role in conjugation (see Table S6). Meanwhile, three repeats of *tet*(X4) were also found in pRF108-1_107k_tetX_flye, but only one *tet*(X4) copy was found in pRF108-2_97k_tetX, indicating that polymorphism of tandem repeats happened during bacterial division of the same clone. The resistome in a single sample was investigated previously (6, 28), but the *tet*(X4)-bearing plasmidome was not studied. Our initial attempt to recover different *tet*(X4)-bearing plasmids in a single microbiota was successful and proved the complex transmission routes of *tet*(X4) among environmental and fecal microbiota. This method is limited because of the number (only two in this study) of *tet*(X4)-bearing strains isolated from one sample; more colonies harboring *tet*(X4) should be recovered and analyzed phenotypically and genomically, and a long-read metagenomics method could be performed to analyze the polymorphism of *tet*(X4)-bearing structures in single-microbiota samples.

10.1128/mSystems.00134-20.8 TABLE S5 Basic information on the seven samples detected with two *tet*(X)-positive strains each. Download Table S5, DOCX file, 0.02 MB. Copyright © 2020 Li et al. 2020 Li et al. This content is distributed under the terms of the Creative Commons Attribution 4.0 International license.

10.1128/mSystems.00134-20.9 TABLE S6 Plasmids participate in reorganization in the process of conjugation. Download Table S6, DOCX file, 0.02 MB. Copyright © 2020 Li et al. 2020 Li et al. This content is distributed under the terms of the Creative Commons Attribution 4.0 International license.

### Co-occurrence of two different *tet*(X) variants in a single sample.

**FIG 5:**
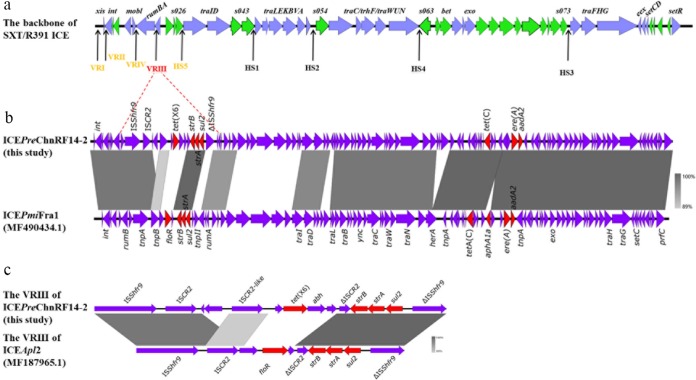
Structures of the *tet*(X6)-bearing ICE and other ICEs found in Providencia rettgeri strain RF14-2 and other bacteria. (a) Typical structure of the SXT/R391 ICE; (b) comparative analysis of the structures of ICEPreChnRF14-2 and ICEPmiFra1; (c) genetic structure of *tet*(X6) and a similar genetic context.

Within the seven samples positive for two *tet*(X)-bearing isolates, strains RF14-1 and RF14-2 belonged to E. coli and Providencia rettgeri, respectively (see Table S5). A *tet*(X4) variant carried by an IncX1 plasmid, with a single nucleotide mutation compared with the *tet*(X4) gene (30), was found in strain RF14-1. This single-base substitution had no effect on the function of the *tet*(X4) gene, which was confirmed by measuring the MICs of E. coli harboring the cloning vector via the TA cloning method. Also, the IncX1-type plasmid carrying the *tet*(X4)-like gene could be successfully transferred into E. coli C600. The other two IncX1 plasmids carrying *tet*(X4) isolated in this study have no transferability (see Table S3). Further comparative analysis of two types of IncX1 plasmids was performed. The plasmids were subsequently analyzed using the Web-based tool oriTfinder to identify the vital elements related to conjugation (51), which showed that pRF14-1_50k_tetX had a complete module pertaining to conjugation which was lacking in pRB3-1_31k_tetX and pRF45-1_31k_tetX (Fig. 4). In Providencia rettgeri strain RF14-2, a novel *tet*(X) variant, designated *tet*(X6), was detected in an SXT/R391 integrative and conjugative element (ICE). The function of *tet*(X6), i.e., the ability to confer tigecycline resistance, was confirmed by the TA cloning method. It was homologous to *tet*(X4) (87.3%) and other *tet*(X) variants (36). Coincidentally, R391 was also first discovered in a Providencia rettgeri clinical isolate (52) and was later classified as the SXT/R391 family (53–55). The ICE in strain RF14-2 integrated into the 5′ end of the gene *prfC*, which was a typical characteristic of all members of the SXT/R391 family (55, 56). This novel SXT/R391 ICE was designated ICE*Pre*ChnRF14-2 according to the nomenclature system (54). Structural analysis showed that the *tet*(X6)-containing region was integrated into variable region III of the ICE (Fig. 5a). In addition, the ICE in RF14-2 had a structure similar to that of ICE*Pmi*Fra1 in Proteus mirabilis in the NCBI database (Fig. 5a). The *tet*(X6)-bearing structure was in the variable region III of the ICE, sharing a similar structure with other ICEs with different accessory regions characterized by IS*CR2* (Fig. 5b). Although the prevalence of the *tet*(X6)-bearing ICE was low compared with the high incidence of *tet*(X4)-bearing plasmids among *tet*(X)-bearing strains in this study, the existence of a *tet*(X6)-bearing ICE located on the chromosome implied that *tet*(X) transmission occurred via multiple routes. The co-occurrence of different *tet*(X) variants in different genetic backgrounds of diverse bacteria highlighted the complex *tet*(X) evolution in microbiotas.

### The diversity of *tet*(X4)-harboring contexts and tandem repeats.

**FIG 6:**
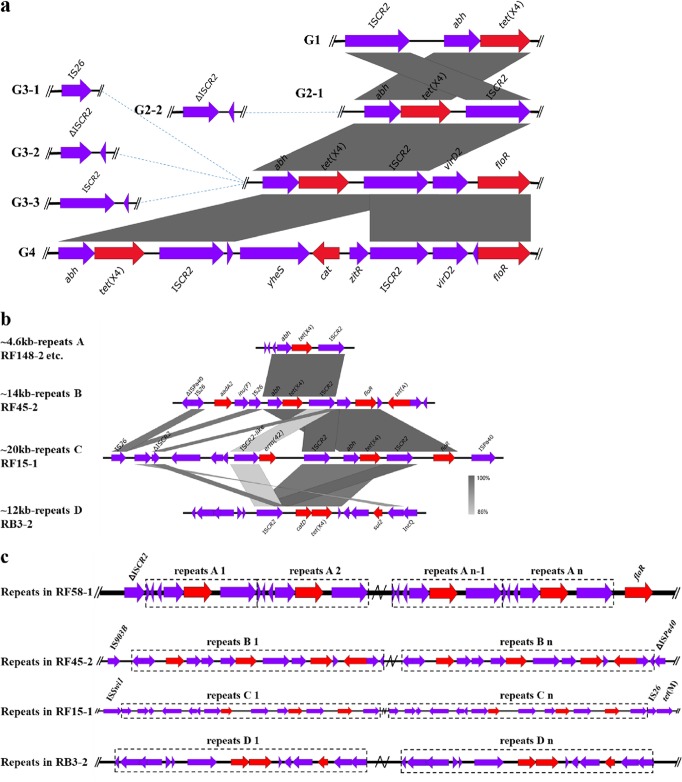
Characterization of *tet*(X4)-bearing genetic contexts. (a) Major types of *tet*(X4)-bearing genetic contexts among the 42 *tet*(X4)-bearing plasmids; (b) four types of *tet*(X4)-bearing tandem repeats; (c) copy number variations of *tet*(X4)-bearing tandem repeat structures based on Nanopore analysis of single molecules.

All the *tet*(X4)-harboring genetic contexts from *tet*(X4)-bearing plasmids in the online database and in this study were analyzed and categorized into four major groups (Fig. 6a). IS*CR2*-*tet*(X4)-*abh* was the prominent structure observed in *tet*(X4)-bearing plasmids (see Table S5). The second type (G2), which had the reverse gene arrangement compared with the first type (G1), was divided into two subtypes depending on the presence of △IS*CR2* (Fig. 6a). The third type (G3), which had the conserved structure *abh*-*tet*(X4)-IS*CR2*-*virD2*-*floR*, was categorized into three subtypes with different genes—IS*26*, △IS*CR2*, and IS*CR2*—in the upstream region. The last genetic structure type (G4) had the longest genetic region, *abh*-*tet*(X4)-IS*CR2*-*yheS*-*cat*-*zitR*-IS*CR2*-*virD2*-*floR*. The first structure type was mainly distributed in small plasmids, including IncQ and ColE2-like plasmids (see Table S4) (31, 57). Distribution of other *tet*(X4)-bearing structures existed in plasmids of different replicons and chromosomes (30, 34), without direct relationship to the *tet*(X4)-bearing structures.

Although Nanopore long-read data were generated to perform *de novo* assembly of *tet*(X4)-bearing plasmids, it was still impossible to obtain complete plasmid sequences for some *tet*(X4)-bearing plasmids, even with different assembly strategies (see Table S3). After single-plasmid-molecule analysis had been performed as described previously (58), all the uncompleted plasmids were found to harbor multiple copies of *tet*(X4). For *tet*(X4)-bearing plasmids with low copy numbers of *tet*(X4), Nanopore long reads could cover the repeat region, and complete plasmid sequences could be finished, such as those for pRF108-1_107k_tetX_flye and pRW8-2_117k_tetX_flye. However, for *tet*(X4)-bearing plasmids with *tet*(X4) tandem repeat regions larger than long reads, or with heterogenous numbers of *tet*(X4) tandem repeats, accurate copy numbers were difficult to obtain, resulting in failure of plasmid assembly. This demonstrated that the repeat regions containing *tet*(X4) were diverse and in a polymorphic state. To summarize, four kinds of repeat regions were detected, which ranged from 4.6 to 20 kb in length (Fig. 6b). They were widely distributed in different types of plasmids. The most common repeat structure was *abh*-*tet*(X4)-IS*CR2* in 4,606 bp, which was the reported *tet*(X4)-bearing circular intermediate (4,608 bp) (30, 43). This circular intermediate may play an important role in the generation of *tet*(X4) tandem repeat structures. An IncQ *tet*(X4)-bearing plasmid, pRB3-2_un_11k_tetX_flye in RB3-2, similar to pLHM10-1-p6 (31), was found in tandem repeats of the whole plasmid, and there could be as many as five repeats after analysis of all the long reads (see Fig. S2). This phenomenon was similar to the reported *tet*(X4)-bearing ColE2-like plasmid p16EC-9K, which was also observed in a polymorphic state of tandem plasmid repeats (57), and this may benefit the transmission of *tet*(X4) (59). Two additional large-repeat structures, IS*26*-*aadA2*-*lnu*(F)-IS*26*-*abh*-*tet*(X4)-IS*CR2*-*floR*-*tet*(A)-△IS*26* in 14 kb and △IS*CR2*-*erm*(42)-IS*CR2*-*abh*-*tet*(X4)-IS*CR2*-*floR*-IS*26* in 20 kb, were found in RF45-2 and RF15-1, respectively. Certain long reads harboring tandem repeats of *tet*(X4) were illustrated to infer the complex structures (see Fig. S2). The 20-kb genetic structure was the longest *tet*(X4)-bearing tandem repeat, implying the complex structures of *tet*(X4) among plasmids. Although the multiple repeats of *tet*(X4) were common in the samples, the MICs of tetracycline, including tigecycline, were not affected significantly. The reason for the frequent occurrence of *tet*(X4) repeats in natural isolates, compared with the low prevalence of duplications of other resistance genes, warrants further investigations.

10.1128/mSystems.00134-20.2 FIG S2 Three major *tet*(X4)-bearing tandem repeat structures resolved by Nanopore long reads. The three parts represent the three types of *tet*(X4)-bearing repeats observed in two Nanopore long reads for each sample. Download FIG S2, JPG file, 0.3 MB. Copyright © 2020 Li et al. 2020 Li et al. This content is distributed under the terms of the Creative Commons Attribution 4.0 International license.

Recently, the polymorphism of *tet*(X4), especially the tandem repeats of the IS*CR2*-*tet*(X4) structure, probably generated by rolling-circle transposition, in E. coli was reported (57). Similarly, IS*CR1*-*qnrB6* tandem repeats following a complex class I integron were also observed in the plasmidome of *Salmonella* and were resolved by single long-read analysis (58). Furthermore, the relative copy numbers of IS*CR2* to *tet*(X4) based on WGS data were analyzed, and the results showed that IS*CR2* copy numbers were equal to or higher than that of *tet*(X4) (Fig. 1), enhancing the idea that IS*CR2* is a pivotal element facilitating *tet*(X4) transmission. The observation of multiple copy numbers of *tet*(X4) in this study suggests that IS*CR* elements may play important roles in the enrichment of resistance genes in microbiota.

### Reorganizations of *tet*(X4)-bearing plasmids.

**FIG 7:**
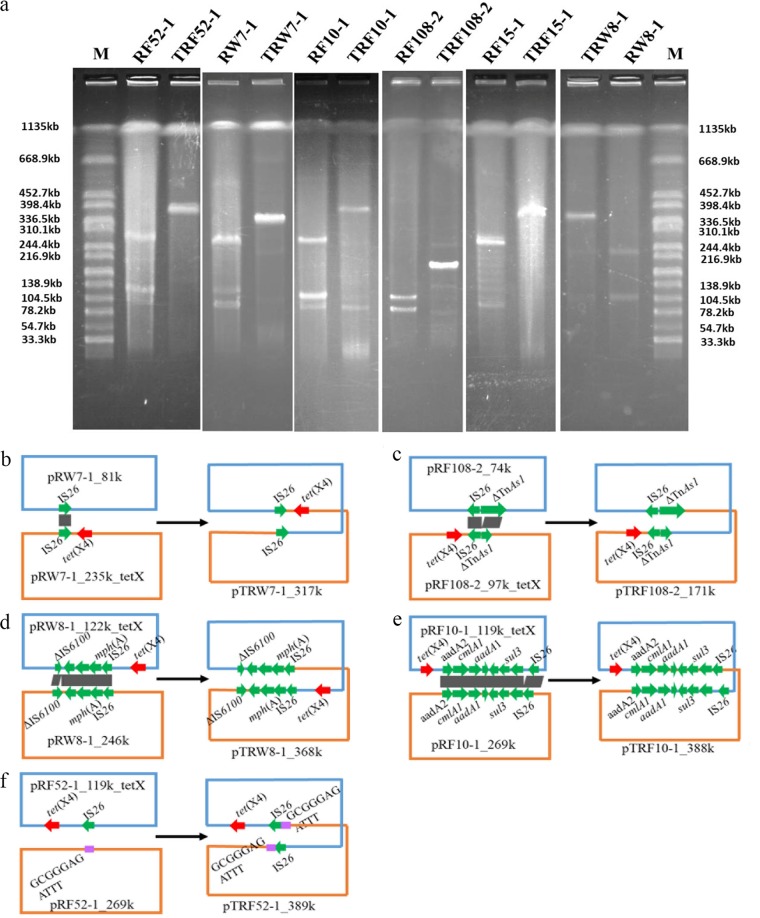
Reorganization of *tet*(X4)-bearing plasmids resolved by S1-PFGE and the underlying molecular mechanisms. (a) S1-PFGE of donor strains and the corresponding transconjugants with plasmid reorganizations (M, molecular weight markers); (b to f) schematic diagrams depicting the generation process of five cointegrate plasmids mediated by homologous regions or IS*26*. Red arrows indicate the *tet*(X4) gene, and green arrows represent the genes involved in plasmid reorganizations. Target site duplications are shown with purple rectangles. The plasmid reorganization of samples RF15-1 and TRF15-1 was not resolved successfully here.

Among the conjugative plasmids, the *tet*(X4)-bearing plasmids of six transconjugants showed different plasmid sizes compared with that of their parental strains after conjugation (Fig. 7a). From the results of previous studies (50, 60, 61), we speculated that the plasmids of these six transconjugants were formed via recombination in the process of conjugation. To probe the molecular mechanism of plasmid reorganization among the six *tet*(X4)-bearing strains, plasmid DNA was extracted from the six transconjugants, and long-read sequencing was performed. All the plasmids were finished in complete and circular forms except RF15-1 and TRF15-1 (see Table S6). Linear comparison between plasmid pTRW7-1_317k in transconjugant TRW7-1 and its parental plasmids in RW7-1 showed that pTRW7-1_317k was derived from the fusion of pRW7-1_235k_tetX and pRW7-1_81k by homologous recombination of IS*26* (Fig. 7b). In TRF108-2, plasmid pTRF108-2_171k was composed of pRF108-2_97k_tetX and pRF8-1_74kb in donor strain RF8-1 generated by homologous recombination of IS*26*-ΔTn*As1* (Fig. 7c). The plasmid pTRW8-1_368k in TRW8-1 was formed by homologous recombination of pRW8-1_246k and pRW8-1_122k_tetX through the genetic structure IS*26*-*mph*(A)-*orf*-*orf*-ΔIS*6100* (Fig. 7d). Similarly, plasmid pTRF10-1_388k was generated by homologous recombination of pRF10-1_269kb and pRF10-1_119k_tetX via the common region IS*26*-*orf*-*sul3*-*orf*-*orf*-*aadA1*-*cmlA1*-*aadA2* (Fig. 7e). Distinctively, the generation of pTRF52-1-389kb was generated after interplasmid (pRF52-1_119k_tetX and pRF52-1_269kb) transposition via IS*26* replicative transposition, resulting in duplications of IS*26* and the target sequence in the cointegrate pTRF52-1_389kb (Fig. 7f). The role of IS*26* in reorganizing plasmids by replicative transposition was recognized in other plasmids among MDR bacteria (62). This was the first report of *tet*(X4)-bearing plasmid reorganization by replicative transposition. For the plasmid reorganization in TRF15-1, the plasmids were not successfully assembled even with the Nanopore long-read data, which implies that possible underlying complex structures exist, and this warrants further investigation.

The *tet*(X4)-bearing plasmid reorganization would incorporate more resistance genes and replicon genes in the novel large MDR hybrid or cointegrate plasmids, which expanded the host range, causing a severe public health concern. Although the reorganizations were observed during conjugation under laboratory conditions, the prevalence of such MDR hybrid or cointegrate plasmids in the natural environment, with a focus on the MDR plasmidome among different environments, should be investigated.

### Conclusions.

The data presented in this study expand the understanding of diversity of *tet*(X4)-bearing plasmids, *tet*(X4)-bearing genetic contexts, and complex *tet*(X4)-bearing plasmid reorganization and highlight the wide distribution of various *tet*(X4)-bearing structures in different E. coli clones. Identification of *tet*(X6)-bearing ICEs in the same *tet*(X4)-positive microbiota spotlighted the existence of coevolution of multiple *tet*(X) variants. Nanopore long reads significantly enhanced the characterization of polymorphism of *tet*(X4)-bearing plasmids from the perspective of single-plasmid-molecule analysis. The importance of plasmids and IS*CR2* in facilitating the transmission of *tet*(X) was confirmed. In summary, this work demonstrates the significant role of the *tet*(X)-bearing plasmidome in the swine slaughterhouse for *tet*(X) transmission along the pork production chain, and stringent surveillance of *tet*(X)-bearing microbiotas of animals, humans, and the environment should be conducted to evaluate the risk posed by the emerging plasmid-mediated tigecycline-resistant *tet*(X) variants.

## MATERIALS AND METHODS

### Sample collection and bacterial isolation.

In May 2019, 240 samples consisting of 182 swine fecal samples, 22 swine carcass samples, 11 ground blood samples, 10 wastewater samples, and 15 soil samples were collected from a slaughterhouse in Jiangsu Province, China. The samples were stored at low temperature during rapid transfer to our lab for further processing. Solid and liquid samples (1 g each) or cotton swabs (surface samples) were incubated in 5 ml LB broth supplemented with tigecycline (2 mg/liter) for 6 h to enrich the tigecycline-resistant microbiota. The enriched cultures were streaked onto MacConkey agar plates containing tigecycline (2 mg/liter) to screen single colonies, and colonies with different morphology characteristics were further purified and stored in LB broth with 15% glycerol at −80°C. Genomic DNA of samples was prepared by the boiling method. The presence of *tet*(X) was checked using PCR with the primers *tet*(X)-F (5′-TGA ACC TGG TAA GAA GAA GTG-3′) and *tet*(X)-R (5′-CAG ACA ATA TCA AAG CAT CCA-3′) (581 bp). The PCR amplicons were subsequently sequenced by Sanger sequencing. 16S rRNA gene sequencing was performed to confirm species identification of the *tet*(X)-positive isolates using universal primers (16S-F, 5′-AGA GTT TGA TCA TGG CTC-3′; 16S-R, 5′-GGT TAC CTT GTT ACG ACT T-3′).

### Antimicrobial susceptibility testing.

The MICs of colistin and tigecycline were determined by the broth microdilution method in accordance with Clinical and Laboratory Standards Institute (CLSI) guidelines (63) and were interpreted according to the European Committee on Antimicrobial Susceptibility Testing (EUCAST) guidelines, with the resistance breakpoint at >2 mg/liter for tigecycline (http://www.eucast.org/clinical_breakpoints/) (64). The MICs of other antimicrobials for the *tet*(X)-positive isolates were measured by the agar microdilution method and interpreted in accordance with the CLSI standard (63). E. coli ATCC 25922 was used as the quality control.

### Filter mating assay and S1-PFGE.

To investigate the transferability of *tet*(X)-carrying genetic elements, a conjugation assay with a filter mating method (65) was carried out using *tet*(X)-positive strains as the donor strains and rifampin-resistant E. coli C600 as the recipient (1:4). Transconjugants were selected on LB agar plates supplemented with rifampin (300 mg/liter) and tigecycline (2 mg/liter). The transconjugants harboring *tet*(X4) were confirmed by PCR and antimicrobial susceptibility testing as described above. To characterize the *tet*(X)-bearing plasmid profiles, *tet*(X)-positive strains and their transconjugants were digested with S1 nuclease (Takara, Osaka, Japan) followed by pulsed-field gel electrophoresis (PFGE) with the CHEF Mapper XA system (Bio-Rad, CA). The *Salmonella* Braenderup H9812 standard strain restricted with XbaI was used as the molecular marker.

### Genome extractions, plasmid extractions, and high-throughput sequencing.

Genomic DNA of the tigecycline-resistant strains was extracted using the TIANamp bacterial DNA kit (TianGen, Beijing, China), following the manufacturer’s instruction. The plasmids of strains were extracted using the Qiagen plasmid midi-kit (Qiagen, Germany) after overnight culture in 100 ml LB broth. The genomic DNA of selected strains with different resistance phenotypes was subjected to short-read sequencing (2 × 150 bp) with the Illumina HiSeq 2500 platform. Subsequently, genomic DNA of certain strains and plasmid DNA were sequenced with the Oxford Nanopore Technologies MinION long-read platform with the RBK004 barcoding library preparation kit and MinION R9.4.1 flow cells to obtain the complete sequences, as described previously (66, 67).

### Bioinformatics analysis.

Short-read Illumina raw sequences of 27 strains were separately assembled using SPAdes (68), and contigs less than 500 bp were discarded. Multilocus sequence typing (MLST) of strains was performed using the mlst tool (https://github.com/tseemann/mlst) based on assembled contigs. The draft genomes were annotated using the software Prokka (69). The phylogenetic tree of these E. coli strains was constructed using Roary and FastTree based on SNPs of core genomes (70, 71). The Flye long-read assembly tool was used to perform *de novo* assembly of Nanopore long-read MinION sequences of plasmids DNA and genomic DNA (72). The draft plasmid and *tet*(X4)-bearing structures were analyzed using the BLASTn program against the nr database. Genomic DNA with short-read Illumina and long-read Nanopore data were used to perform *de novo* assembly with the hybrid strategy as described previously (67). The high-quality complete genome sequences were annotated using RAST (http://rast.nmpdr.org/) automatically and modified manually. Plasmid replicons, insertion sequences, and antimicrobial resistance determinants were determined using online tools (https://cge.cbs.dtu.dk/services/). BRIG and Easyﬁg were used to generate the genetic comparison ﬁgures (73, 74). All *tet*(X4)-bearing plasmids available in the NCBI nr database were downloaded for further analysis. The diversity and heterogeneous status of the *tet*(X4)-bearing plasmidome were investigated based on the Nanopore single-molecule analysis method (58).

### Data availability.

The *tet*(X4)-bearing plasmids generated in this study were deposited in the National Center for Biotechnology Information database (see Table S4). The assembled plasmid sequences with only Nanopore data and single long reads analyzed individually were deposited in the figshare database (https://figshare.com/s/6077f70a0ec952ee2796) for reference. Other data that support the findings of this research are available upon request.

10.1128/mSystems.00134-20.3 FIG S3 Linear comparisons between *tet*(X4)-bearing plasmids in transconjugants and their parental plasmids in donor strains to visualize molecular mechanisms of plasmid reorganizations among the five *tet*(X4)-bearing strains. The gray regions denote the homologous regions between the plasmids. The red arrow signifies the *tet*(X4) gene, and blue indicates gene clusters involved in plasmid reorganization. Download FIG S3, JPG file, 2.6 MB. Copyright © 2020 Li et al. 2020 Li et al. This content is distributed under the terms of the Creative Commons Attribution 4.0 International license.
