# Supplementary material for: Deciphering the Structural Diversity and Classification of the Mobile Tigecycline Resistance Gene tet(X)-Bearing Plasmidome among Bacteria
Source: mSystems. 2020 Apr 28;5(2):e00134-20. doi: 10.1128/mSystems.00134-20 (PMC7190383; doi:10.1128/mSystems.00134-20)
Supplement: TABLE S1 [file mSystems.00134-20-st001.docx]

**Supplementary Table 1. Numbers of *tet*(X4) positive strains and their prevalence among different sources in the slaughterhouse.**

| Sources | Number of samples | Number of *tet*(X4) positive strains | Number of positive samples | Positive rates^a^ |
| --- | --- | --- | --- | --- |
| soil | 15 | 8 | 6 | 40.00% |
| blood | 11 | 4 | 3 | 27.27% |
| wastewater | 10 | 5 | 4 | 40.00% |
| carcass | 22 | 2 | 2 | 9.09% |
| faeces | 182 | 56 | 53 | 29.12% |
| total | 240 | 75 | 68 | 28.33% |

^a^ Positive rate= Number of positive samples/Number of samples.
