# Supplementary material for: Deciphering the Structural Diversity and Classification of the Mobile Tigecycline Resistance Gene tet(X)-Bearing Plasmidome among Bacteria
Source: mSystems. 2020 Apr 28;5(2):e00134-20. doi: 10.1128/mSystems.00134-20 (PMC7190383; doi:10.1128/mSystems.00134-20)
Supplement: TABLE S2 [file mSystems.00134-20-st002.docx]

**Supplementary Table 2. Antibiotic susceptibility testing (mg/L) of 74 *E. coli and* 1 *Providencia rettgeri* harboring the *tet*(X4) or *tet*(X6) genes.**

| IDs^a^ | Sources | Species | Antimicrobials | | | | | | | | | | | | |
| --- | --- | --- | --- | --- | --- | --- | --- | --- | --- | --- | --- | --- | --- | --- | --- |
|  |  |  | TIG | CL | AMX | CQM | MEM | DOX | ENR | ATM | FFC | TET | CFF | STR | KAN |
| RF10-1 | faeces | *E．coli* | 8 | ≤0.125 | >256 | 8 | ≤0.125 | 32 | >32 | 2 | >128 | >64 | >64 | >64 | 4 |
| RF106-1 | faeces | *E．coli* | 8 | ≤0.125 | >256 | ≤0.125 | ≤0.125 | 32 | 0.5 | ≤0.125 | >128 | >64 | 4 | 4 | 4 |
| RF107-1 | faeces | *E．coli* | 8 | 0.5 | >256 | 0.25 | ≤0.125 | 64 | 32 | ≤0.125 | >128 | >64 | 8 | 32 | 4 |
| RF109-1 | faeces | *E．coli* | 8 | ≤0.125 | >256 | ≤0.125 | ≤0.125 | 32 | 16 | ≤0.125 | >128 | >64 | >64 | >64 | 32 |
| RF113-1 | faeces | *E．coli* | 16 | ≤0.125 | >256 | ≤0.125 | ≤0.125 | 64 | 1 | ≤0.125 | >128 | >64 | 4 | 4 | 4 |
| RF115-1 | faeces | *E．coli* | 16 | ≤0.125 | >256 | 2 | ≤0.125 | 64 | 0.5 | 0.5 | >128 | >64 | >64 | 32 | >64 |
| RF116-1 | faeces | *E．coli* | 16 | ≤0.125 | >256 | ≤0.125 | ≤0.125 | 64 | >64 | ≤0.125 | >128 | >64 | 16 | >64 | 4 |
| RF12-1 | faeces | *E．coli* | 16 | ≤0.125 | >256 | ≤0.125 | ≤0.125 | 64 | 0.5 | ≤0.125 | >128 | >64 | 4 | >64 | 4 |
| RF123-1 | faeces | *E．coli* | 16 | ≤0.125 | >256 | 4 | ≤0.125 | 64 | >32 | 0.5 | >128 | >64 | >64 | >64 | 4 |
| RF125-1 | faeces | *E．coli* | 16 | ≤0.125 | >256 | ≤0.125 | ≤0.125 | 64 | 0.5 | ≤0.125 | >128 | >64 | 4 | 4 | 4 |
| RF133-1 | faeces | *E．coli* | 8 | ≤0.125 | >256 | 1 | ≤0.125 | 64 | 0.5 | 0.5 | >128 | >64 | >64 | 32 | >64 |
| RF138-1 | faeces | *E．coli* | 8 | ≤0.125 | >256 | ≤0.125 | ≤0.125 | 64 | 2 | ≤0.125 | >128 | >64 | 2 | 64 | 4 |
| RF144-1 | faeces | *E．coli* | 8 | ≤0.125 | >256 | ≤0.125 | ≤0.125 | 64 | 0.25 | 0.5 | >128 | >64 | >64 | 32 | >64 |
| RF146-1 | faeces | *E．coli* | 16 | ≤0.125 | >256 | ≤0.125 | ≤0.125 | 64 | 0.25 | 2 | >128 | >64 | >64 | >64 | >64 |
| RF15-1 | faeces | *E．coli* | 16 | ≤0.125 | >256 | ≤0.125 | ≤0.125 | 64 | >32 | 2 | >128 | >64 | >64 | >64 | 4 |
| RF154-1 | faeces | *E．coli* | 16 | ≤0.125 | >256 | ≤0.125 | ≤0.125 | 64 | >32 | 2 | >128 | >64 | >64 | >64 | 4 |
| RF155-1 | faeces | *E．coli* | 8 | ≤0.125 | >256 | ≤0.125 | ≤0.125 | 32 | >64 | ≤0.125 | >128 | >64 | 8 | >64 | 4 |
| RF157-1 | faeces | *E．coli* | 16 | ≤0.125 | >256 | 2 | ≤0.125 | 64 | 8 | 1 | >128 | >64 | >64 | >64 | 8 |
| RF162-1 | faeces | *E．coli* | 8 | ≤0.125 | >256 | ≤0.125 | ≤0.125 | 64 | >32 | 1 | >128 | >64 | >64 | 32 | >64 |
| RF168-1 | faeces | *E．coli* | 16 | ≤0.125 | >256 | 2 | ≤0.125 | 64 | 0.25 | 0.5 | >128 | >64 | >64 | 64 | >64 |
| RF169-1 | faeces | *E．coli* | 16 | ≤0.125 | >256 | ≤0.125 | ≤0.125 | 64 | 0.25 | ≤0.125 | 128 | >64 | 2 | >64 | 2 |
| RF178-1 | faeces | *E．coli* | 16 | ≤0.125 | >256 | ≤0.125 | ≤0.125 | 64 | 0.25 | ≤0.125 | >128 | >64 | >64 | >64 | >64 |
| RF180-1 | faeces | *E．coli* | 8 | ≤0.125 | >256 | 1 | ≤0.125 | 32 | 16 | 0.5 | >128 | >64 | >64 | >64 | 16 |
| RF2-1 | faeces | *E．coli* | 16 | ≤0.125 | >256 | 0.25 | ≤0.125 | 32 | 1 | ≤0.125 | >128 | >64 | 4 | 8 | >64 |
| RF23-1 | faeces | *E．coli* | 16 | ≤0.125 | >256 | ≤0.125 | ≤0.125 | 64 | >32 | 2 | >128 | >64 | >64 | >64 | 4 |
| RF25-1 | faeces | *E．coli* | 8 | ≤0.125 | >256 | 2 | ≤0.125 | 64 | 4 | 0.5 | >128 | >64 | >64 | >64 | 16 |
| RF30-1 | faeces | *E．coli* | 8 | ≤0.125 | >256 | 2 | ≤0.125 | 64 | 0.5 | 0.5 | >128 | >64 | >64 | 32 | >64 |
| RF3-1 | faeces | *E．coli* | 16 | ≤0.125 | >256 | ≤0.125 | ≤0.125 | 32 | >32 | ≤0.125 | >128 | >64 | 2 | >64 | 4 |
| RF36-1 | faeces | *E．coli* | 8 | ≤0.125 | >256 | ≤0.125 | ≤0.125 | 64 | 1 | ≤0.125 | >128 | >64 | 8 | 4 | 4 |
| RF40-1 | faeces | *E．coli* | 8 | ≤0.125 | >256 | ≤0.125 | ≤0.125 | 32 | 16 | ≤0.125 | >128 | >64 | 2 | 32 | 4 |
| RF47-1 | faeces | *E．coli* | 16 | ≤0.125 | >256 | ≤0.125 | ≤0.125 | 32 | 2 | ≤0.125 | >128 | >64 | 4 | 4 | 4 |
| RF48-1 | faeces | *E．coli* | 16 | ≤0.125 | >256 | 2 | ≤0.125 | 64 | 1 | 2 | >128 | >64 | >64 | >64 | >64 |
| RF52-1 | faeces | *E．coli* | 8 | ≤0.125 | >256 | 4 | ≤0.125 | 32 | >32 | 2 | >128 | >64 | >64 | >64 | 4 |
| RF58-1 | faeces | *E．coli* | 16 | ≤0.125 | >256 | ≤0.125 | ≤0.125 | 64 | 0.25 | 0.25 | >128 | >64 | 4 | 16 | 16 |
| RF6-1 | faeces | *E．coli* | 8 | ≤0.125 | >256 | ≤0.125 | ≤0.125 | 32 | 0.25 | ≤0.125 | >128 | >64 | 4 | 4 | 4 |
| RF62-1 | faeces | *E．coli* | 8 | ≤0.125 | >256 | 1 | ≤0.125 | 32 | 32 | ≤0.125 | >128 | >64 | >64 | >64 | >64 |
| RF65-1 | faeces | *E．coli* | 8 | ≤0.125 | >256 | ≤0.125 | ≤0.125 | 32 | 0.5 | ≤0.125 | >128 | >64 | 4 | 16 | 16 |
| RF67-1 | faeces | *E．coli* | 16 | ≤0.125 | >256 | ≤0.125 | ≤0.125 | 64 | 1 | ≤0.125 | >128 | >64 | 1 | 2 | 2 |
| RF71-1 | faeces | *E．coli* | 16 | ≤0.125 | >256 | ≤0.125 | ≤0.125 | 64 | 1 | ≤0.125 | >128 | >64 | 4 | 16 | 16 |
| RF73-1 | faeces | *E．coli* | 16 | ≤0.125 | >256 | 2 | ≤0.125 | 32 | 0.125 | 0.25 | >128 | >64 | >64 | 32 | >64 |
| RF76-1 | faeces | *E．coli* | 16 | ≤0.125 | 4 | 4 | ≤0.125 | 32 | 0.25 | ≤0.125 | >128 | >64 | 1 | 2 | 4 |
| RF80-1 | faeces | *E．coli* | 16 | 0.25 | >256 | ≤0.125 | ≤0.125 | 64 | 2 | ≤0.125 | >128 | >64 | 8 | 4 | 4 |
| RF83-1 | faeces | *E．coli* | 16 | ≤0.125 | >256 | ≤0.125 | ≤0.125 | 64 | 0.25 | 1 | >128 | >64 | >64 | 32 | >64 |
| RF94-1 | faeces | *E．coli* | 8 | ≤0.125 | >256 | ≤0.125 | ≤0.125 | 64 | 1 | ≤0.125 | >128 | >64 | 2 | 32 | >64 |
| RF98-1 | faeces | *E．coli* | 16 | ≤0.125 | >256 | ≤0.125 | ≤0.125 | 64 | 0.25 | ≤0.125 | >128 | >64 | 1 | 4 | 4 |
| RF108-1 | faeces | *E．coli* | 16 | ≤0.125 | >256 | 0.5 | ≤0.125 | 64 | 0.25 | ≤0.125 | 128 | >64 | 1 | 2 | 4 |
| RF108-2 | faeces | *E．coli* | 8 | ≤0.125 | >256 | 1 | ≤0.125 | 64 | 1 | 0.25 | >128 | >64 | 64 | 16 | >64 |
| RF173-1 | faeces | *E．coli* | 8 | ≤0.125 | >256 | ≤0.125 | ≤0.125 | 32 | 32 | ≤0.125 | >128 | >64 | 8 | 32 | 4 |
| RF55-1 | faeces | *E．coli* | 16 | ≤0.125 | 4 | 4 | ≤0.125 | 32 | 0.25 | ≤0.125 | >128 | >64 | 1 | 4 | 4 |
| RF148-1 | faeces | *E．coli* | 16 | ≤0.125 | >256 | ≤0.125 | ≤0.125 | 32 | >32 | ≤0.125 | >128 | >64 | 2 | >64 | 2 |
| RF148-2 | faeces | *E．coli* | 8 | ≤0.125 | >256 | ≤0.125 | ≤0.125 | 32 | 1 | ≤0.125 | >128 | >64 | 8 | 4 | 4 |
| RF14-1 | faeces | *E．coli* | 8 | ≤0.125 | >256 | ≤0.125 | ≤0.125 | 32 | 0.5 | ≤0.125 | >128 | >64 | 1 | 4 | >64 |
| RF14-2 | faeces | *Providencia rettgeri* | 16 | >64 | 256 | ≤0.125 | ≤0.125 | >128 | 1 | ≤0.125 | >128 | >64 | 16 | 16 | >64 |
| RF37-1 | faeces | *E．coli* | 8 | ≤0.125 | 4 | 128 | ≤0.125 | 32 | 0.25 | ≤0.125 | 128 | >64 | 8 | 4 | >64 |
| RF45-1 | faeces | *E．coli* | 16 | ≤0.125 | >256 | ≤0.125 | ≤0.125 | 64 | 0.5 | 0.25 | 128 | >64 | 64 | 32 | >64 |
| RF45-2 | faeces | *E．coli* | 16 | ≤0.125 | >256 | 1 | ≤0.125 | 128 | 0.5 | 0.25 | >128 | >64 | >64 | 64 | >64 |
| RB8-1 | blood | *E．coli* | 32 | 0.25 | >256 | 2 | ≤0.125 | 128 | 0.25 | 0.5 | >128 | >64 | >64 | 32 | >64 |
| RB9-1 | blood | *E．coli* | 8 | ≤0.125 | >256 | 1 | ≤0.125 | 64 | 2 | ≤0.125 | 64 | >64 | >64 | >64 | >64 |
| RB3-1 | blood | *E．coli* | 32 | ≤0.125 | >256 | 4 | ≤0.125 | 64 | 0.25 | 0.25 | >128 | >64 | >64 | >64 | >64 |
| RB3-2 | blood | *E．coli* | 8 | ≤0.125 | >256 | ≤0.125 | ≤0.125 | 64 | 2 | ≤0.125 | >128 | >64 | 8 | >64 | 8 |
| RS3-1 | soil | *E．coli* | 16 | ≤0.125 | >256 | ≤0.125 | ≤0.125 | 64 | 2 | ≤0.125 | >128 | >64 | 8 | 16 | >64 |
| RS3-2 | soil | *E．coli* | 8 | ≤0.125 | 256 | ≤0.125 | ≤0.125 | 64 | 0.5 | ≤0.125 | >128 | >64 | 8 | >64 | 4 |
| RS2-1 | soil | *E．coli* | 16 | ≤0.125 | >256 | ≤0.125 | ≤0.125 | 32 | 0.5 | ≤0.125 | >128 | >64 | 2 | >64 | 4 |
| RS5-1 | soil | *E．coli* | 16 | ≤0.125 | >256 | 1 | ≤0.125 | 128 | 0.25 | 0.25 | >128 | >64 | >64 | 64 | >64 |
| RS6-1 | soil | *E．coli* | 8 | ≤0.125 | >256 | ≤0.125 | ≤0.125 | 8 | 1 | ≤0.125 | 64 | >64 | 8 | 4 | 4 |
| RS6-2 | soil | *E．coli* | 8 | ≤0.125 | >256 | ≤0.125 | ≤0.125 | 64 | 1 | ≤0.125 | >128 | >64 | 8 | >64 | 4 |
| RS5-1 | soil | *E．coli* | 16 | ≤0.125 | >256 | 1 | ≤0.125 | 64 | 0.25 | ≤0.125 | >128 | >64 | 4 | 4 | 4 |
| RS8-1 | soil | *E．coli* | 8 | ≤0.125 | >256 | ≤0.125 | ≤0.125 | 32 | 0.5 | ≤0.125 | >128 | >64 | 4 | 32 | 4 |
| RW4-1 | wastewater | *E．coli* | 8 | ≤0.125 | >256 | ≤0.125 | ≤0.125 | 32 | 0.5 | ≤0.125 | >128 | >64 | 2 | 2 | 4 |
| RW6-1 | wastewater | *E．coli* | 8 | ≤0.125 | >256 | ≤0.125 | ≤0.125 | 64 | 0.5 | ≤0.125 | >128 | >64 | 16 | 32 | 4 |
| RW7-1 | wastewater | *E．coli* | 16 | 0.25 | >256 | ≤0.125 | ≤0.125 | 64 | 4 | ≤0.125 | >128 | >64 | 8 | >64 | >64 |
| RW8-1 | wastewater | *E．coli* | 8 | ≤0.125 | >256 | 4 | ≤0.125 | 32 | >32 | 2 | >128 | >64 | >64 | >64 | 4 |
| RW8-2 | wastewater | *E．coli* | 8 | 0.5 | >256 | ≤0.125 | ≤0.125 | 32 | 1 | ≤0.125 | >128 | >64 | 8 | 32 | 4 |
| RT18-1 | carcass | *E．coli* | 16 | ≤0.125 | 128 | ≤0.125 | ≤0.125 | 32 | 16 | ≤0.125 | >128 | >64 | 8 | >64 | 2 |
| RT29-1 | carcass | *E．coli* | 8 | ≤0.125 | 256 | ≤0.125 | ≤0.125 | 64 | 0.5 | ≤0.125 | 64 | >64 | 4 | >64 | 2 |
| ATCC25922 | NA | *E．coli* | 0.125 | ≤0.125 | 2 | ≤0.125 | ≤0.125 | 0.25 | ≤0.125 | ≤0.125 | 4 | 1 | 0.5 | 4 | 4 |

**Abbreviations**: TIG, tigecycline; CL, colistin; AMX, amoxicillin; CQM, cefquinome; MEM, meropenem; DOX, doxycycline; ENR, enrofloxacin; ATM, aztreonam; FFC, florfenicol; TET, tetracycline; CFF, ceftiofur; STR, streptomycin; KAN, kanamycin.

^a^ The strains with the same IDs before the dash symbol were from the samples. Seven samples were recovered with two *tet*(X) positive strains respectively.
