# Supplementary material for: Deciphering the Structural Diversity and Classification of the Mobile Tigecycline Resistance Gene tet(X)-Bearing Plasmidome among Bacteria
Source: mSystems. 2020 Apr 28;5(2):e00134-20. doi: 10.1128/mSystems.00134-20 (PMC7190383; doi:10.1128/mSystems.00134-20)
Supplement: TABLE S3 [file mSystems.00134-20-st003.docx]

**Supplementary Table 3. Basic information of 27 *tet*(X4)-bearing plasmids in this study.**

| Plasmids | Status^a^ | Size(bp) | Conjugative | Inc-type | *tet*(X4) repeats |
| --- | --- | --- | --- | --- | --- |
| pRB3-1_31k_tetX | complete | 31287 | - | IncX1 | one |
| pRF14-1_50k_tetX | complete | 50518 | + |  | one |
| pRF45-1_31k_tetX | complete | 31287 | - |  | one |
| pRF45-2_un_65k_tetX_flye | incomplete | 65547 | - |  | multiple |
| pRF10-1_119k_tetX | complete | 119011 | + | IncFIB(K), IncFIA(HI1), IncX1 | one |
| pRF108-1_107k_tetX_flye | complete | 107701 | - |  | three |
| pRF108-2_97k_tetX | complete | 97526 | + |  | one |
| pRF52-1_119k_tetX | complete | 119180 | + |  | one |
| pRF148-1_119k_tetX | complete | 119185 | - |  | one |
| pRF148-2_101k_tetX | complete | 101373 | - |  | one |
| pRW8-1_122k_tetX | complete | 122608 | + |  | one |
| pRW8-2_117k_tetX_flye | complete | 117983 | - |  | two |
| pRF76-1_un_105k_tetX_flye | incomplete | 105990 | + |  | multiple |
| pRF155_129k_tetX_flye | complete | 129122 | + |  | one |
| pRF15-1_un_207k_tetX_flye | incomplete | 207400 | + |  | multiple |
| pRF173-2_87k_tetX | complete | 87445 | + | IncA/C2 | one |
| pRS3-1_136k_tetX_flye | complete | 136546 | + | IncFIB(AP001918) | one |
| pRS3-2_194k_tetX_flye | complete | 194164 | - | IncFIA(HI1), IncHI1A, IncHI1B(R27) | one |
| pRW7-1_235k_tetX | complete | 235947 | + | IncFIA(HI1), IncHI1A, IncHI1B(R27), IncX1 | one |
| pRS6-2_229k_tetX_flye | complete | 229965 | - |  | one |
| pRT18-1_294k_tetX | complete | 294397 | - | IncFIB(K), IncFIA(HI1), IncHI1A, IncHI1B(R27) | one |
| pRF25-1_12k_tetX_flye | complete | 12888 | + | IncQ1 | one |
| pRB3-2_un_11k_tetX_flye | incomplete | 11480 | - |  | multiple |
| pRF58-1_un_136k_tetX_flye | incomplete | 136918 | + | IncFⅡ | multiple |
| pRF2-1_117k_tetX_flye | complete | 117635 | + |  | one |
| pRF65-1_113k_tetX_flye | complete | 113072 | + |  | one |
| pRF71-1_112k_tetX_flye | complete | 112916 | + |  | one |

^a^ Plasmid with incomplete status indicates the sequences are not circular due to the multiple repeats of *tet*(X4)-bearing genetic structures.
