# Supplementary material for: Deciphering the Structural Diversity and Classification of the Mobile Tigecycline Resistance Gene tet(X)-Bearing Plasmidome among Bacteria
Source: mSystems. 2020 Apr 28;5(2):e00134-20. doi: 10.1128/mSystems.00134-20 (PMC7190383; doi:10.1128/mSystems.00134-20)
Supplement: TABLE S4 [file mSystems.00134-20-st004.docx]

**Supplementary Table 4. Basic information of 42 *tet*(X4)-bearing plasmids in NCBI nr database and this study.**

| Accession No.^a^ | Definition | Assembly  Method | Sequencing Technology | Submission date | Plasmids | Resistance genes | Replicons | *tet*(X4) genetic contexts^b^ | Plasmid length | Isolation source | Country | References (DOI) |
| --- | --- | --- | --- | --- | --- | --- | --- | --- | --- | --- | --- | --- |
| CP046004 | *Escherichia coli* strain 1919D3 plasmid p1919D3-1, complete sequence | A5-miseeq; CANU | Illumina MiSeq; PacBio | 08-NOV-2019 | p1919D3-1 | *aadA1*, *aadA2*, *aadA22*, *tet*(A), *tet*(X4), *sul3*, *lnu*(G), *bla*_TEM-1B_, *dfrA12*, *cmlA1*, *floR* | IncFIA(HI1),IncHI1A,IncHI1B(R27) | G4 | 219101 bp | Swine faeces | China: Henan | Not Available |
| CP046007 | *Escherichia coli* strain 1919D62 plasmid p1919D62-1, complete sequence | A5-miseeq; CANU | Illumina MiSeq; PacBio | 08-NOV-2019 | p1919D62-1 | *tet*(X4), *lnu*(G), *floR*, *qnrS1*, *bla*_TEM-1B_, *aadA22* | IncFIA(HI1),IncHI1A,IncHI1B(R27) | G2-1 | 186058 bp | Swine faeces | China: Henan | Not Available |
| CP046002 | *Escherichia coli* strain 1916D6 plasmid p1916D6-2, complete sequence | A5-miseeq; CANU | Illumina MiSeq; PacBio | 08-NOV-2019 | p1916D6-2 | *tet*(A), *tet*(X4), *floR*, *aadA2*, *lnu*(F), *bla*_SHV-12_ | IncX1 | G3-1 | 59351 bp | Swine faeces | China: Henan | Not Available |
| CP045998 | *Escherichia coli* strain 1916D18 plasmid p1916D18-1, complete sequence | A5-miseeq; CANU | Illumina MiSeq; PacBio | 08-NOV-2019 | p1916D18-1 | *aadA2*, *tet*(A), *tet*(X4), *bla*_SHV-12_, *floR*, *lnu*(F) | IncX1 | G3-1 | 59353 bp | Swine faeces | China: Henan | Not Available |
| CP041453 | *Escherichia coli* strain YPE3 plasmid pYPE3-92k-tetX4, complete sequence | Unicycler | Oxford Nanopore MinION, Illumina | 09-JUL-2019 | pYPE3-92k-tetX4 | *mef*(B), *floR*, *sul3*, *tet*(X4), *dfrA5* | IncFIA(HI1),IncFIB(K),IncX1 | G4 | 92973 bp | Pork | China: Yangzhou | 10.1093/jac/dkz528 |
| CP041449 | *Escherichia coli* strain YPE10 plasmid pYPE10-190k-tetX4, complete sequence | Unicycler | Oxford Nanopore MinION, Illumina | 09-JUL-2019 | pYPE10-190k-tetX4 | *floR*, *tet*(X4), *bla*_TEM-1B_, *lnu*(G), *aadA22,* *qnrS1* | IncFIA(HI1), IncHI1A, IncHI1B(R27) | G4 | 190128 bp | Pork | China: Yangzhou | 10.1093/jac/dkz528 |
| CP041443 | *Escherichia coli* strain YPE12 plasmid pYPE12-101k-tetX4, complete sequence | Unicycler | Oxford Nanopore MinION, Illumina | 09-JUL-2019 | pYPE12-101k-tetX4 | *mef*(B), *tet*(A), *tet*(M), *tet*(X4), *floR*, *dfrA5,* *bla*_TEM-1B_, *qnrS1*, *sul3* | IncFIA(HI1), IncFIB(K), IncX1 | G4 | 101987 bp | Pork | China: Yangzhou | 10.1093/jac/dkz528 |
| MN101858 | *Escherichia coli* strain 2019XSD11-TC2 plasmid p2019XSD11-TC2-284, complete sequence | RS HGAP assembly;  Unicycler | nanopore; PacBio | 23-JUN-2019 | p2019XSD11-TC2-284 | *aadA22*, *strA*, *strB*, *bla*_CTX-M-55_, *bla*_TEM-1B_, *floR*, *lnu*(G), *qnrS1*, *fosA3*, *tet*(A), *tet*(X4), *sul2* | IncFIA(HI1), IncFII(pHN7A8), IncHI1A, IncHI1B(R27) | G4 | 284309 bp | Laboratory strain | China | Not Available |
| MN101856 | *Escherichia coli* strain 2019XSD11 plasmid p2019XSD11-190, complete sequence | RS HGAP assembly; Unicycler | nanopore; PacBio | 23-JUN-2019 | p2019XSD11-190 | *qnrS1*, *bla*_TEM-1B_, *floR*, *aadA22*, *lnu*(G), *tet*(X4) | IncFIA(HI1)，IncHI1A，IncHI1B(R27) | G4 | 190390 bp | Pork | China | Not Available |
| MK134376 | *Escherichia coli* strain 47EC plasmid p47EC, complete sequence | HGAP | PacBio | 06-NOV-2018 | p47EC | *qnrS1*, *erm*(42), *lnu*(F), *floR*, *bla*_TEM-1B_, *sul3*, *aadA1*, *aadA2b*, *aph(3')-Ia*, *tet*(A),,*tet*(M), *tet*(X4) | IncFIB (AP001918), IncFIB(K) | G2-2 | 170312 bp | Pig fecal swab | China | 10.1038/s41564-019-0445-2 |
| CP040929 | *Escherichia coli* strain YY76-1 plasmid pYY76-1-2, complete sequence | canu | Oxford Nanopore GridION | 10-JUN-2019 | pYY76-1-2 | *tet*(A), *tet*(X4), *bla*_SHV-12_, *aadA2*, *floR*, *lnu*(F) | IncX1 | G3-1 | 57104 bp | Cow faeces | China: Guangdong | doi: 10.1128/AAC.01528-19 |
| CP037909 | *Escherichia coli* strain LHM10-1 plasmid pLHM10-1, complete sequence | Unicycler | PacBio | 12-MAR-2019 | pLHM10-1 | *tet*(X4) | IncQ1 | G1 | 12783 bp | Pig fecal | China: Jiangxi | doi: 10.1038/s41564-019-0496-4 |
| CP037911 | *Escherichia coli* strain YSP8-1 plasmid pYSP8-1, complete sequence | Unicycler | PacBio | 12-MAR-2019 | pYSP8-1 | *aadA22*, *floR,* *erm*(B), *lnu*(G), *tet*(X4) *bla*_TEM-1B_, *qnrS1* | IncFIA(HI1)，IncHI1A，IncHI1B(R27) ，IncX4 | G3-3 | 239511 bp | Pig fecal | China: Guangdong | 10.1038/s41564-019-0496-4 |
| CP038140 | *Escherichia coli* strain G3X16-2 plasmid pG3X16-2-3, complete sequence | Unicycler | Oxford Nanopore GridION | 21-MAR-2019 | pG3X16-2-3 | *bla*_TEM-1B_, *sul3*, *cmlA1*, *floR*, *erm*(42), *dfrA12*, *tet*(A), *tet*(M), *tet*(X4), *aadA1*, *aadA2*, *strA*, *strB* | IncFIA(HI1)，IncFIB(K)，IncX1 | G3-3 | 138950 bp | Pig fecal | China: Guangxi | 10.1038/s41564-019-0496-4 |
| MN381965 | *Escherichia coli* strain 16EC plasmid p16EC-9K, complete sequence | Unicycler | Oxford Nanopore Technologies (ONT) MinION | 26-AUG-2019 | p16EC-9K | *tet*(X4) | - | G1 | 9228  bp | pig | China | 10.1128/AAC.01825-19 |
| MT197111 | *Escherichia coli* strain RB3-1 plasmid pRB3-1_31k_tetX, complete sequence | Unicycler | Oxford Nanopore MinION, Illumina | 28-DEC-2019 | pRB3-1_31k_tetX | *floR*, *aadA2*, *tet(*A), *tet*(X4), *lnu*(F) | IncX1 | G3-1 | 31287 bp | blood | China: Jiangsu | This study |
| figshare | *Escherichia coli* strain RB3-2 plasmid pRB3-2_un_11k_tetX_flye, complete sequence | flye | Oxford Nanopore MinION, | 28-DEC-2019 | pRB3-2_un_11k_tetX_flye | *tet*(X4) | IncQ1 | G1 | 11480 bp | blood | China: Jiangsu | This study |
| MT219822 | *Escherichia coli* strain RF14-1 plasmid pRF14-1_50k_tetX, complete sequence | Unicycler | Oxford Nanopore MinION, Illumina | 19-MAR-2020 | pRF14-1_50k_tetX | *bla*_TEM-176_, *aph(3')-Ia*, *qnrS1*, *tet*(X4) | IncX1 | G2-1 | 50518 bp | Swine faeces | China: Jiangsu | This study |
| MT219821 | *Escherichia coli* strain RF45-1 plasmid pRF45-1_31k_tetX, complete sequence | Unicycler | Oxford Nanopore MinION, Illumina | 19-MAR-2020 | pRF45-1_31k_tetX | *floR*, *aadA2*, *tet*(A), *tet*(X4), *lnu*(F) | IncX1 | G3-1 | 31287 bp | Swine faeces | China: Jiangsu | This study |
| figshare | *Escherichia coli* strain RF45-2 plasmid pRF45-2_un_65k_tetX_flye, complete sequence | flye | Oxford Nanopore MinION | 28-Dec-2019 | pRF45-2_un_65k_tetX_flye | *floR*, *aadA2*, *tet*(A), *tet*(X4), *lnu*(F) | IncX1 | G3-1 | 65547 bp | Swine faeces | China: Jiangsu | This study |
| MT219823 | *Escherichia coli* strain RF10-1 plasmid pRF10-1_119k_tetX, complete sequence | Unicycler | Oxford Nanopore MinION, Illumina | 19-MAR-2020 | pRF10-1_119k_tetX | *erm*(42), *aadA1*, *aadA2*, *strA, strB*, *dfrA12*, *tet*(A), *tet*(M), *tet*(X4), *cmlA*, *floR*, *sul3* | IncFIB(K)，IncFIA(HI1)，IncX1 | G3-3 | 119011 bp | Swine faeces | China: Jiangsu | This study |
| figshare | *Escherichia coli* strain RF108-1 plasmid pRF108-1_107k_tetX_flye, complete sequence | flye | Oxford Nanopore MinION | 28-DEC-2019 | pRF108-1_107k_tetX_flye | *dfrA5*, *floR*, *qnrS1*, *bla*_TEM-1B_, *mef*(B), *tet*(A), *tet*(M), *tet*(X4), *sul3* | IncFIB(K)，IncFIA(HI1)，IncX1 | G3-2 | 107701 bp | Swine faeces | China: Jiangsu | This study |
| MT219820 | *Escherichia coli* strain RF108-2 plasmid pRF108-2_97k_tetX, complete sequence | Unicycler | Oxford Nanopore MinION, Illumina | 19-MAR-2020 | pRF108-2_97k_tetX | *mef*(B), *bla*_TEM-1B_, *qnrS1*, *tet*(A), *tet*(M), *tet*(X4), *sul3*, *floR*, *dfrA5* | IncFIB(K)，IncFIA(HI1)，IncX1 | G3-2 | 97526 bp | Swine faeces | China: Jiangsu | This study |
| MT219819 | *Escherichia coli* strain RF52-1 plasmid pRF52-1_119k_tetX, complete sequence | Unicycler | Oxford Nanopore MinION, Illumina | 19-MAR-2020 | pRF52-1_119k_tetX | *tet*(A), *tet*(M), *tet*(X4), *erm*(42), *cmlA1*, *floR*, *dfrA12*, *aadA1*, *aadA2*, *strA, strB*, *sul3* | IncFIB(K)，IncFIA(HI1)，IncX1 | G3-3 | 119180 bp | Swine faeces | China: Jiangsu | This study |
| MT219818 | *Escherichia coli* strain RF148-1 plasmid pRF148-1_119k_tetX, complete sequence | Unicycler | Oxford Nanopore MinION, Illumina | 19-MAR-2020 | pRF148-1_119k_tetX | *mph*(A), *cmlA1*, *floR*, *bla*_TEM-1B_, *dfrA12*, *tet*(A), *tet*(M), *tet*(X4), *sul3*, *aadA1*, *aadA2*, *strA, strB* | IncFIB(K)，IncFIA(HI1)，IncX1 | G3-2 | 119185 bp | Swine faeces | China:Jiangsu | This study |
| MT219817 | *Escherichia coli* strain RF148-2 plasmid pRF148-2_101k_tetX, complete sequence | Unicycler | Oxford Nanopore MinION, Illumina | 19-MAR-2020 | pRF148-2_101k_tetX | *bla*_TEM-1B_, *qnrS1*, *floR,* *tet*(A), *tet*(M), *tet*(X4), *dfrA5*, *mef*(B), *sul3* | IncFIB(K)，IncFIA(HI1)，IncX1 | G3-2 | 101373 bp | Swine faeces | China:Jiangsu | This study |
| MT219826 | *Escherichia coli* strain RW8-1 plasmid pRW8-1_122k_tetX, complete sequence | Unicycler | Oxford Nanopore MinION, Illumina | 19-MAR-2020 | pRW8-1_122k_tetX | *aadA1*, *aadA2*, *strA, strB*, *bla*_TEM-1B_, *sul3*, *mph*(A), *dfrA12*, *tet*(A), *tet*(M), *tet*(X4), *cml*(A1), *floR* | IncFIB(K)，IncFIA(HI1)，IncX1 | G2-1 | 122608 bp | Wastewater | China: Jiangsu | This study |
| figshare | *Escherichia coli* strain RW8-2 plasmid pRW8-2_117k_tetX_flye, complete sequence | flye | Oxford Nanopore MinION | 28-Dec-2019 | pRW8-2_117k_tetX_flye | *tet*(A), *tet(*M), *tet*(X4), *qnrS1*, *aadA1*, *bla*_TEM-1B_, *sul2*, *floR* | IncFIB(K)，IncFIA(HI1)，IncX1 | G2-2 | 117983 bp | Wastewater | China: Jiangsu | This study |
| figshare | *Escherichia coli* strain RF76-1 plasmid pRF76-1_un_105k_tetX_flye, complete sequence | flye | Oxford Nanopore MinION | 28-DEC-2019 | pRF76-1_un_105k_tetX_flye | *sul3*, *mef*(B), *floR*, *tet*(A), *tet*(M), *tet*(X4), *qnrS1*, *dfrA5* | IncFIB(K)，IncFIA(HI1)，IncX1 | G3-3 | 105990 bp | Swine faeces | China: Jiangsu | This study |
| figshare | *Escherichia coli* strain RF155-1 plasmid pRF155-1_129k_tetX_flye, complete sequence | flye | Oxford Nanopore MinION | 28-DEC-2019 | pRF155-1_129k_tetX_flye | *tet*(A), *tet*(M), *tet*(X4), *dfrA12*, *erm*(42), *mph*(A), *bla*_TEM-1B_, *cmlA1*, *floR*, *sul3*, *aadA1*, *aadA2*, *strA, strB* | IncFIB(K)，IncFIA(HI1)，IncX1 | G3-3 | 129122 bp | Swine faeces | China:Jiangsu | This study |
| MT219816 | *Escherichia coli* strain RF173-1 plasmid pRF173-1_87k_tetX , complete sequence | Unicycler | Oxford Nanopore MinION, Illumina | 19-MAR-2020 | pRF173-2_87k_tetX | *erm*(42), *tet*(X4) | IncA/C2 | G2-2 | 87445 bp | Swine faeces | China: Jiangsu | This study |
| figshare | *Escherichia coli* strain RS3-1 plasmid pRS3-1_136k_tetX_flye, complete sequence | flye | Oxford Nanopore MinION | 28-DEC-2019 | pRS3-1_136k_tetX_flye | *aadA2b,* *aph(3’)-Ia*, *qnrS1*, *qnrS9*, *erm*(42), *lnu*(F), *sul3*, *floR*, *bla*_TEM-1B_, *tet*(M), *tet*(X4) | IncFIB(AP001918) | G2-2 | 136546 bp | Soil | China: Jiangsu | This study |
| figshare | *Escherichia coli* strain RS3-2 plasmid pRS3-2_194k_tetX_flye, complete sequence | flye | Oxford Nanopore MinION | 28-DEC-2019 | pRS3-2_194k_tetX_flye | *qnrS1*, *aadA22*, *tet*(X4), *floR*, *lnu*(G), *bla*_TEM-1B_ | IncFIA(HI1)， IncHI1A， IncHI1B(R27) | G4 | 194164 bp | Soil | China: Jiangsu | This study |
| MT219825 | *Escherichia coli* strain RW7-1 plasmid pRW7-1_235k_tetX, complete sequence | Unicycler | Oxford Nanopore MinION, Illumina | 19-MAR-2020 | pRW7-1_235k_tetX | *lnu*(G), *bla*_TEM-1B_, *tet*(X4), *floR*, *aadA22*, *aph(3’)-Ia*, *qnrS1*, *qnrS2* | IncFIA(HI1)， IncHI1A， IncHI1B(R27)，IncX1 | G4 | 235947 bp | Wastewater | China: Jiangsu | This study |
| MT219824 | *Escherichia coli* strain RT18-1 plasmid pRT18-1_294k_tetX, complete sequence | Unicycler | Oxford Nanopore MinION, Illumina | 19-MAR-2020 | pRT18-1_294k_tetX | *floR*, *tet*(X4), *lnu*(G), *bla*_TEM-1B_, *aadA22*, *qnrS1* | IncFIB(K)， IncFIA(HI1) ，IncHI1A， IncHI1B(R27) | G2-1 | 294397 bp | carcass | China: Jiangsu | This study |
| figshare | *Escherichia coli* strain RS6-2 plasmid pRS6-2_229k_tetX_flye, complete sequence | flye | Oxford Nanopore MinION | 28-DEC-2019 | pRS6-2_229k_tetX_flye | *lnu*(G), *floR*, *bla*_TEM-1B_, *sul3*, *qnrS1*, *dfrA14*, *tet*(X4), *tet*(A), *aadA22* | IncHI1B(R27)，IncFIA(HI1)，IncHI1A， IncX1 | G4 | 229965 bp | Soil | China: Jiangsu | This study |
| figshare | *Escherichia coli* strain RF25-1 plasmid pRF25-1_12k_tetX_flye, complete sequence | flye | Oxford Nanopore MinION | 28-DEC-2019 | pRF25-1_12k_tetX_flye | *tet*(X4) | IncQ1 | G1 | 12888 bp | Swine faeces | China:Jiangsu | This study |
| figshare | *Escherichia coli* strain RF58-1 plasmid pRF58-1_un_136k_tetX_flye, complete sequence | flye | Oxford Nanopore MinION | 28-DEC-2019 | pRF58-1_un_136k_tetX_flye | *floR*, *sul2*, *qnrS1*, *aac(3)-IId*, *tet*(X4) | IncFⅡ | G3-3 | 136918 bp | Swine faeces | China: Jiangsu | This study |
| figshare | *Escherichia coli* strain RF2-1 plasmid pRF2-1_117k_tetX_flye, complete sequence | flye | Oxford Nanopore MinION | 28-DEC-2019 | pRF2-1_117k_tetX_flye | *tet*(X4), *floR*, *qnrS1*, *erm*(42), *sul2*, *aac(3)-IId* | IncFⅡ | G2-2 | 117635 bp | Swine faeces | China: Jiangsu | This study |
| figshare | *Escherichia coli* strain RF65-1 plasmid pRF65-1_113k_tetX_flye, complete sequence | flye | Oxford Nanopore MinION | 28-DEC-2019 | pRF65-1_113k_tetX_flye | *sul2*, *erm*(42), *qnrS1*, *aac(3)-IId*, *floR,* *tet*(X4) | IncFⅡ | G2-2 | 113072 bp | Swine faeces | China: Jiangsu | This study |
| figshare | *Escherichia coli* strain RF71-1 plasmid pRF71-1_112k_tetX_flye, complete sequence | flye | Oxford Nanopore MinION | 28-DEC-2019 | pRF71-1_112k_tetX_flye | *tet*(X4), *sul2*, *floR*, *aac(3)-IId*, *erm*(42), *qnrS1* | IncFⅡ | G2-2 | 112916 bp | Swine faeces | China: Jiangsu | This study |
| figshare | *Escherichia coli* strain RF15-1 plasmid pRF15-1_un_207k_tetX_flye, complete sequence | flye | Oxford Nanopore MinION | 28-DEC-2019 | pRF15-1_un_207k_tetX_flye | *sul3*, *aadA1*, *aadA2*, *strA, strB*, *dfrA12*, *erm*(42), *cmlA1*, *floR*, *tet*(A), *tet*(M), *tet*(X4) | IncFIA(HI1), IncFIB(K), IncX1 | G3-3 | 207400 bp | Swine faeces | China: Jiangsu | This study |

^a^ The plasmid sequences assembled with only long-read data were submitted in fighshare database (https://figshare.com/s/6077f70a0ec952ee2796).

^b^ The *tet*(X4) genetic context types were linked to the *tet*(X4)-bearing structures illustrated in Fig. 6a.
