# Supplementary figures and images for: Deciphering the Structural Diversity and Classification of the Mobile Tigecycline Resistance Gene tet(X)-Bearing Plasmidome among Bacteria
Source: mSystems. 2020 Apr 28;5(2):e00134-20. doi: 10.1128/mSystems.00134-20 (PMC7190383; doi:10.1128/mSystems.00134-20)

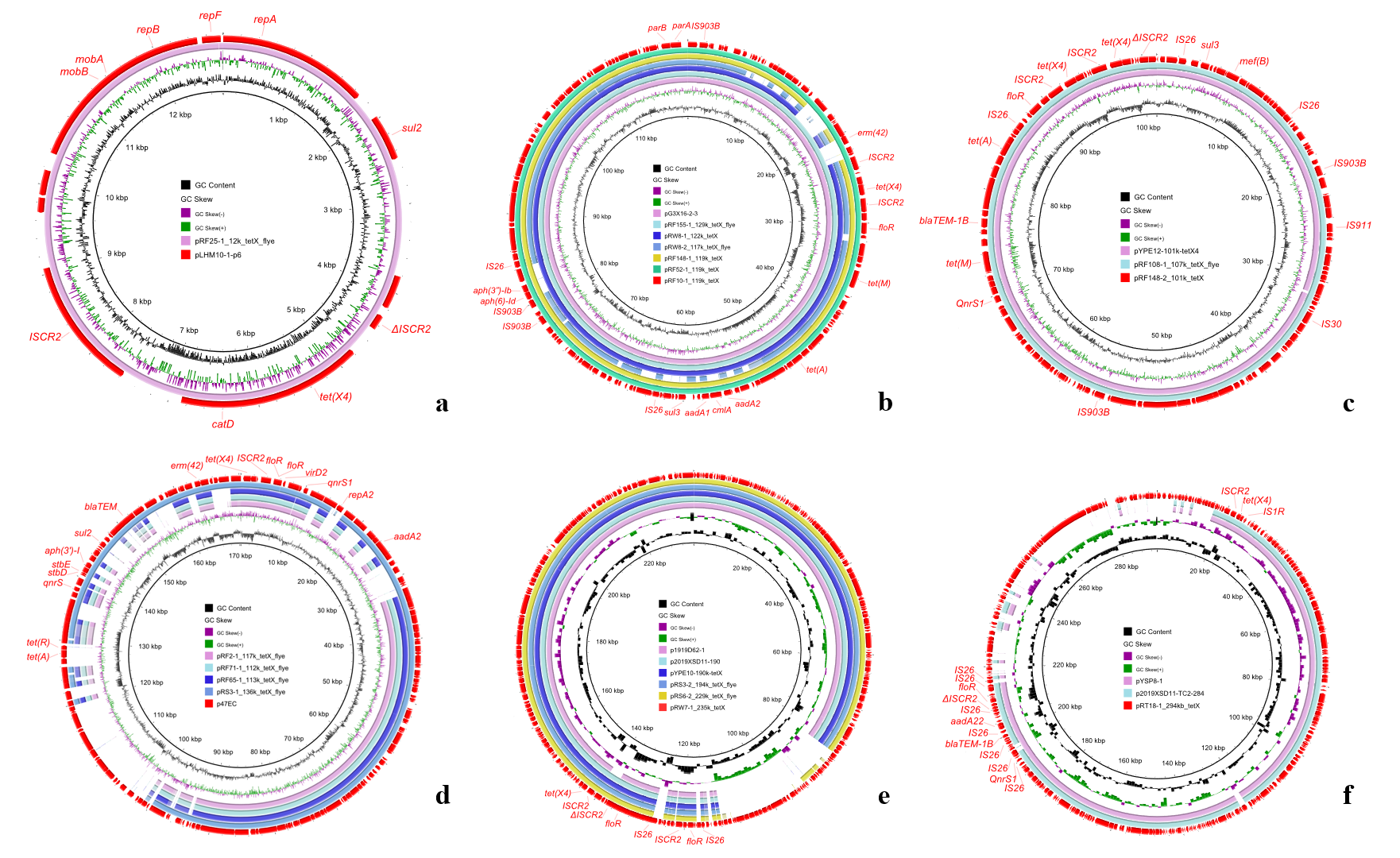

Supplement: FIG S1 [file mSystems.00134-20-sf001.jpg]

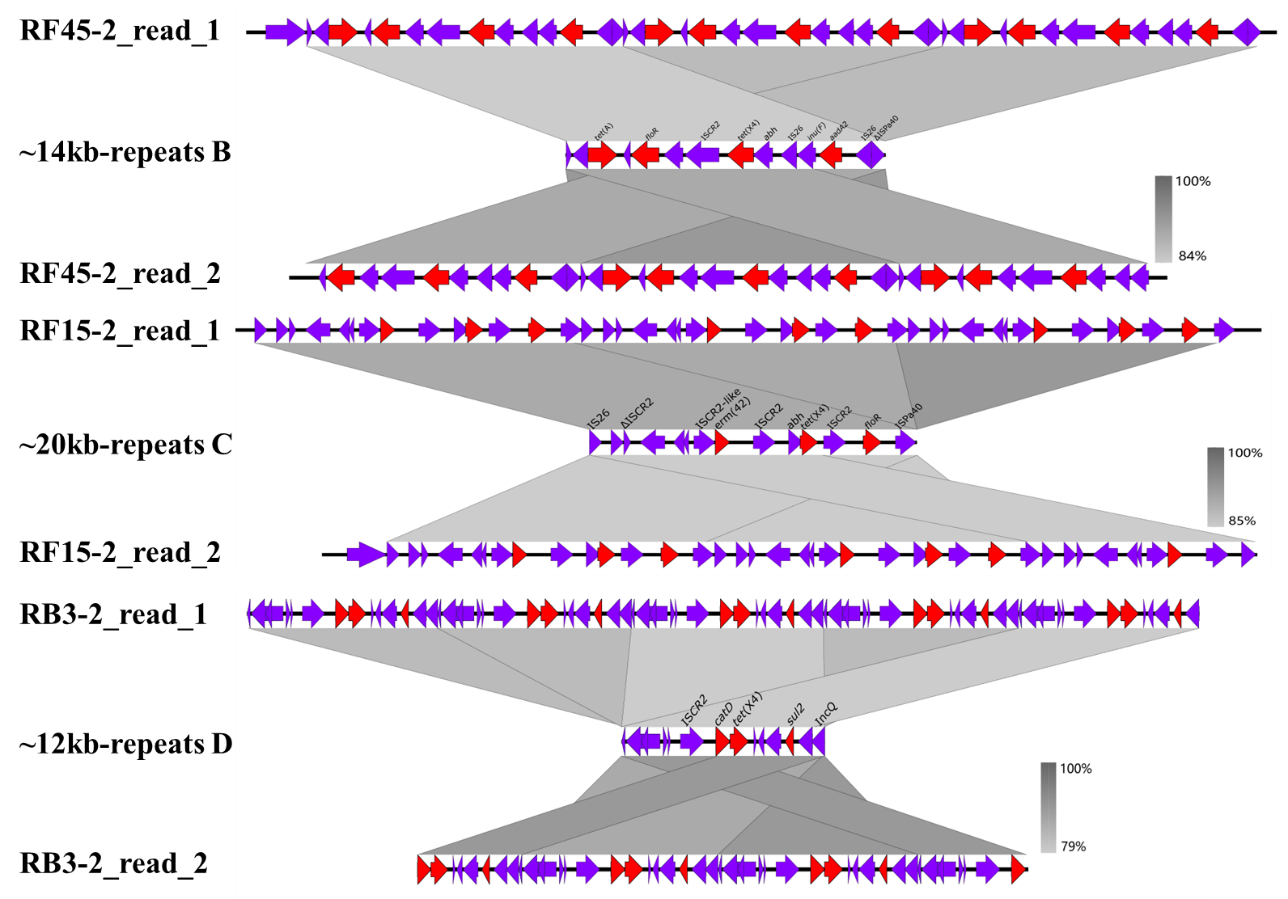

Supplement: FIG S2 [file mSystems.00134-20-sf002.jpg]

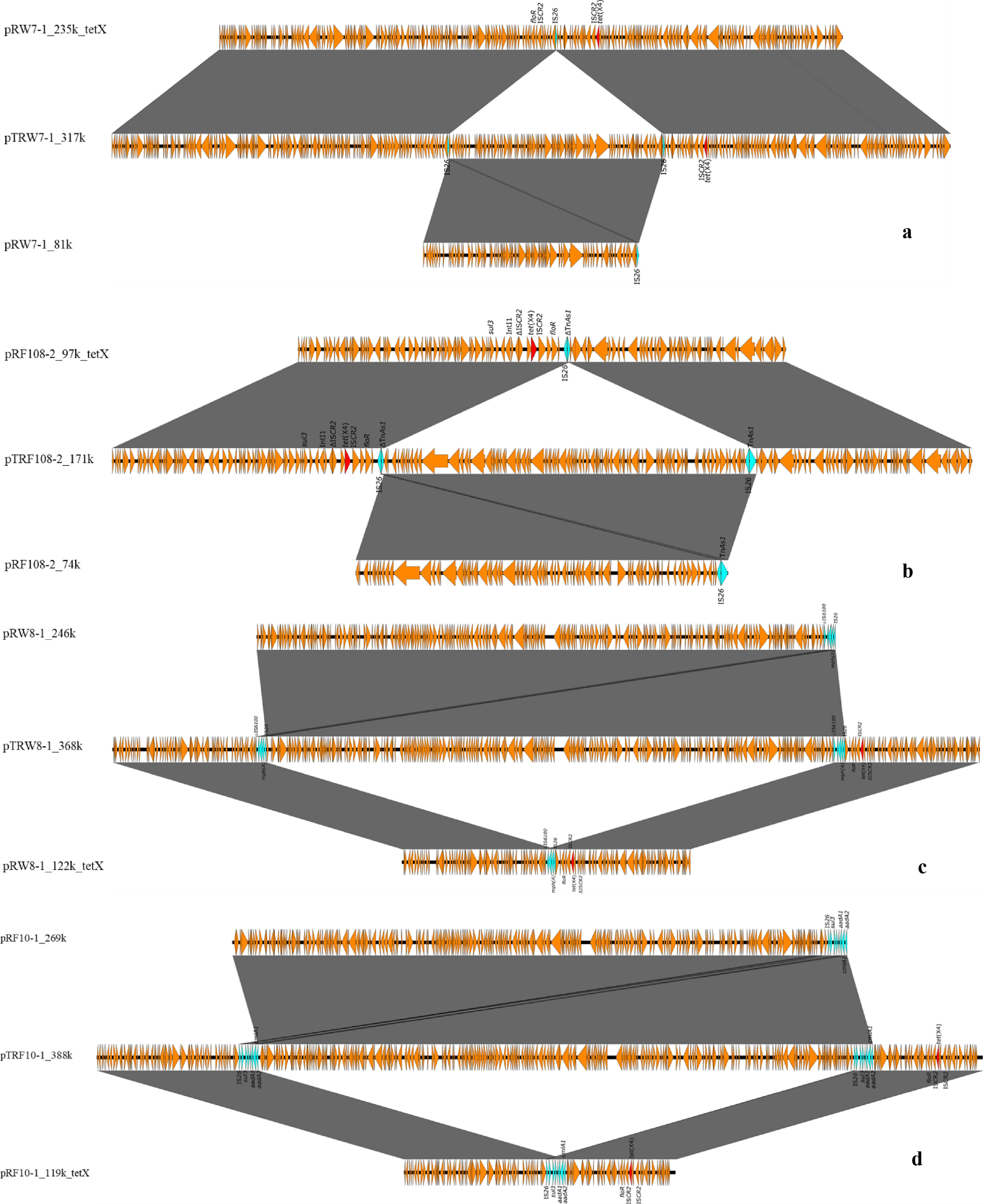

Supplement: FIG S3 [file mSystems.00134-20-sf003.jpg]
