# Supplementary material for: Deciphering the Structural Diversity and Classification of the Mobile Tigecycline Resistance Gene tet(X)-Bearing Plasmidome among Bacteria
Source: mSystems. 2020 Apr 28;5(2):e00134-20. doi: 10.1128/mSystems.00134-20 (PMC7190383; doi:10.1128/mSystems.00134-20)
Supplement: TABLE S5 [file mSystems.00134-20-st005.docx]

**Supplementary Table 5. Basic information of the seven samples detected with two *tet*(X) positive strains.**

| Sample IDs | Source | Strain IDs | ST-type | Species | Assembly  Method | Sequencing Technology | Locations of *tet*(X) | Resistance genes | Replicons | *tet*(X) genetic contexts^a^ | Plasmid length |
| --- | --- | --- | --- | --- | --- | --- | --- | --- | --- | --- | --- |
| RF148 | faeces | RF148-1 | ST1196 | *E. coli* | Unicycler | Oxford Nanopore MinION, Illumina | pRF148-1_119k_tetX | *mph*(A), *cmlA1*, *floR*, *bla*_TEM-1B_, *dfrA12*, *tet*(A), *tet*(M), *tet*(X4), *sul3*, *aadA1*, *aadA2*, *strA, strB* | IncFIB(K)，IncFIA(HI1)，IncX1 | G3-2 | 119185 bp |
|  |  | RF148-2 | ST761 | *E. coli* | Unicycler | Oxford Nanopore MinION, Illumina | pRF148-2_101k_tetX | *bla*_TEM-1B_, *qnrS1*, *floR,* *tet*(A), *tet*(M), *tet*(X4), *dfrA5*, *mef*(B), *sul3* | IncFIB(K)，IncFIA(HI1)，IncX1 | G3-2 | 101373 bp |
| RB3 | blood | RB3-1 | ST195 | *E. coli* | Unicycler | Oxford Nanopore MinION, Illumina | pRB3-1_31k_tetX | *floR*, *aadA2*, *tet(*A), *tet*(X4), *lnu*(F) | IncX1 | G3-1 | 31287 bp |
|  |  | RB3-2 | ST1638 | *E. coli* | flye | Oxford Nanopore MinION, | pRB3-2_un_11k_tetX_flye | *tet*(X4) | IncQ1 | G1 | 11480 bp |
| RW8 | wastewater | RW8-1 | ST1196 | *E. coli* | Unicycler | Oxford Nanopore MinION, Illumina | pRW8-1_122k_tetX | *aadA1*, *aadA2*, *strA, strB*, *bla*_TEM-1B_, *sul3*, *mph*(A), *dfrA12*, *tet*(A), *tet*(M), *tet*(X4), *cml*(A1), *floR* | IncFIB(K)，IncFIA(HI1)，IncX1 | G2-1 | 122608 bp |
|  |  | RW8-2 | ST641 | *E. coli* | flye | Oxford Nanopore MinION | pRW8-2_117k_tetX_flye | *tet*(A), *tet(*M), *tet*(X4), *qnrS1*, *aadA1*, *bla*_TEM-1B_, *sul2*, *floR* | IncFIB(K)，IncFIA(HI1)，IncX1 | G2-2 | 117983 bp |
| RF14 | faeces | RF14-1 | ST10 | *E. coli* | Unicycler | Oxford Nanopore MinION, Illumina | pRF14-1_50k_tetX | *bla*_TEM-176_, *aph(3')-Ia*, *qnrS1*, *tet*(X4) | IncX1 | G2-1 | 50518 bp |
|  |  | RF14-2 | ST | *Providencia rettgeri* | flye | Oxford Nanopore MinION | Chromosome  (ICE*Pre*ChnRF14-2) | *sul2,* *aadA2b,* *strA, strB,* *dfrA32,* *ere*(A)*,* *tet*(C)*,* *tet*(X6) | ICE | -^a^ | - |
| RS3 | soil | RS3-1 | ST-10671 | *E. coli* | flye | Oxford Nanopore MinION | pRS3-1_136k_tetX_flye | *aadA2b,* *aph(3')-Ia*, *qnrS1*, *qnrS9*, *erm*(42), *lnu*(F), *sul3*, *floR*, *bla*_TEM-1B_, *tet*(M), *tet*(X4) | IncFIB(AP001918) | G2-2 | 136546 bp |
|  |  | RS3-2 | ST1638 | *E. coli* | flye | Oxford Nanopore MinION | pRS3-2_194k_tetX_flye | *qnrS1*, *aadA22*, *tet*(X4), *floR*, *lnu*(G), *bla*_TEM-1B_ | IncFIA(HI1), IncHI1A， IncHI1B(R27) | G4 | 194164 bp |
| RF45 | faeces | RF45-1 | ST195 | *E. coli* | Unicycler | Oxford Nanopore MinION, Illumina | pRF45-1_31k_tetX | *floR*, *aadA2*, *tet*(A), *tet*(X4), *lnu*(F) | IncX1 | G3-1 | 31287 bp |
|  |  | RF45-2 | ST195 | *E. coli* | flye | Oxford Nanopore MinION | pRF45-2_un_65k_tetX_flye | *floR*, *aadA2*, *tet*(A), *tet*(X4), *lnu*(F) | IncX1 | G3-1 | 65547 bp |
| RF108 | faeces | RF108-1 | ST761 | *E. coli* | flye | Oxford Nanopore MinION | pRF108-1_107k_tetX_flye | *dfrA5*, *floR*, *qnrS1*, *bla*_TEM-1B_, *mef*(B), *tet*(A), *tet*(M), *tet*(X4), *sul3* | IncFIB(K)，IncFIA(HI1)，IncX1 | G3-2 | 107701 bp |
|  |  | RF108-2 | ST761 | *E. coli* | Unicycler | Oxford Nanopore MinION, Illumina | pRF108-2_97k_tetX | *mef*(B), *bla*_TEM-1B_, *qnrS1*, *tet*(A), *tet*(M), *tet*(X4), *sul3*, *floR*, *dfrA5* | IncFIB(K)，IncFIA(HI1)，IncX1 | G3-2 | 97526 bp |

^a^ The *tet*(X4) genetic context types were linked to the *tet*(X4)-bearing structures illustrated in Fig. 6a. The detailed structure tet(X6)-bearing ICE was presented in Fig. 5.
