# Supplementary material for: Deciphering the Structural Diversity and Classification of the Mobile Tigecycline Resistance Gene tet(X)-Bearing Plasmidome among Bacteria
Source: mSystems. 2020 Apr 28;5(2):e00134-20. doi: 10.1128/mSystems.00134-20 (PMC7190383; doi:10.1128/mSystems.00134-20)
Supplement: TABLE S6 [file mSystems.00134-20-st006.docx]

**Supplementary Table 6. Plasmids participate in reorganization in the process of conjugation.**

| strains | Plasmids participate in Reorganization | Replicon types | Resistance genes |
| --- | --- | --- | --- |
| RF52-1 | pRF52-1_269k | IncHI2, IncHI2A | *aac(3)-IV*, *aadA1*, *aadA2b*, *aph(4)-Ia*, *cmlA1*, *floR*, *bla*_CTX-M-14_, *sul1*, *sul2*, *sul3*, *fosA3* |
|  | pRF52-1_119k_tetX | IncFIA(HI1), IncFIB(K), IncX1 | *tet*(A), *tet*(M), *tet*(X4), *erm*(42), *cmlA1*, *floR*, *dfrA12*, *aadA1*, *aadA2*, *strA, strB*, *sul3* |
| RF10-1 | pRF10-1_269k | IncHI2, IncHI2A | *fosA3*, *sul1*, *sul2*, *sul3*, *aac(3)-IV*, *aadA1*, *aadA2b*, *aph(4)-Ia*, *bla*_CTX-M-14_, *cmlA1*, *floR* |
|  | pRF10-1_119k_tetX | IncFIA(HI1), IncFIB(K), IncX1 | *erm*(42), *aadA1*, *aadA2*, *strA, strB*, *dfrA12*, *tet*(A), *tet*(M), *tet*(X4), *cmlA*, *floR*, *sul3* |
| RW8-1 | pRW8-1_246k | IncHI2, IncHI2A | *fosA3*, *aac(3)-IV*, *aph(4)-Ia*, *erm*(B), *mph*(A), *sul1*, *sul2*, *bla*_CTX-M-14_, *floR*, *oqxA*, *oqxB* |
|  | pRW8-1_122k_tetX | IncFIA(HI1), IncFIB(K), IncX1 | *aadA1*, *aadA2*, *strA, strB*, *bla*_TEM-1B_, *sul3*, *mph*(A), *dfrA12*, *tet*(A), *tet*(M), *tet*(X4), *cml*(A1), *floR* |
| RF108-2 | pRF108-2_97kb_tetX | IncFIA(HI1), IncFIB(K), IncX1 | *mef*(B), *bla*_TEM-1B_, *qnrS1*, *tet*(A), *tet*(M), *tet*(X4), *sul3*, *floR*, *dfrA5* |
|  | pRF108-2_74k | IncFII | *erm*(B), *mph*(A) |
| RW7-1 | pRW7-1_235k_tetX | IncFIA(HI1), IncHI1A, IncHI1B(R27), IncX1 | *lnu*(G), *bla*_TEM-1B_, *tet*(X4), *floR*, *aadA22*, *aph(3')-Ia*, *qnrS1*, *qnrS2* |
|  | pRW7-1_81k | IncFII | *tet*(M), *erm*(B), *mph*(A) |
